# Practicability of Activating Transcription Factor 3 as a Serological Marker for Severity Appraisal and Outcome Anticipation in Acute Supratentorial Intracerebral Hemorrhage: A Two‐center Observational Analytical Study

**DOI:** 10.1002/brb3.71070

**Published:** 2025-11-17

**Authors:** Yingrui Gu, Xin Wang, Peng Xu, Zhiyong Li, Xiaodong Deng, Yong Cui

**Affiliations:** ^1^ Department of Neurosurgery Seventh People's Hospital of Shanghai University of Traditional Chinese Medicine Shanghai China; ^2^ Department of Neurosurgery Shanghai Baoshan Luodian Hospital Baoshan Shanghai China

**Keywords:** ATF3, biomarkers, END, outcome, prognosis, severity, SAP, ICH

## Abstract

**Background:**

Activating transcription factor 3 (ATF3) is a neuroprotective factor and participates in acute brain injury. Here, we intend to determine whether serum ATF3 levels are associated with the severity and clinical outcomes of acute intracerebral hemorrhage (ICH).

**Methods:**

In this two‐center observational analytic study of 236 patients with supratentorial ICH and 114 controls, serum ATF3 levels were quantified at the admission of all patients, at study entry of all controls, and at serial time‐points of 114 patients from all patients. The Admission National Institutes of Health Stroke Scale (NIHSS) and hematoma volume, and the modified Rankin Scale (mRS) at post‐ICH six months were recorded. Outcome variables were stroke‐associated pneumonia (SAP), early neurological deterioration (END), and post‐ICH 6‐month neurological status. Associations were appraised via multifactorial analyses.

**Results:**

Serum ATF3 levels of patients consenting for serial sampling swiftly increased since admission, peaked until post‐ICH day 3, then slowly declined up to day 14, and were significantly higher even on day 14 than those of controls. Dynamic serum ATF3 levels of patients consenting for serial sampling were firmly related to NIHSS scores, hematoma volume, SAP, END, continuous and ordinal mRS scores, and poor prognosis (mRS scores of 3–6). Even adjusting for possible confounding factors, associations of admission serum ATF3 levels with the preceding parameters still existed among all patients. Alternatively, admission serum ATF3 levels were linearly correlated with SAP, END, and poor prognosis under restricted cubic spline, and did not interact with age, sex, alcohol consumption and others via subgroup analysis. The associations were robust in sensitivity analysis. Through receiver operating characteristic (ROC) curve analysis, admission serum ATF3 levels had predictive ability comparable to that of NIHSS scores and hematoma volume.

**Conclusion:**

Elevated serum ATF3 levels following ICH are intimately correlated with disease severity and effectively predict END, SAP, and poor prognosis, solidifying serum ATF3 as a prognostic candidate of ICH.Elevated serum ATF3 levels following ICH are closely correlated with disease severity and effectively predict END, SAP, and poor prognosis, reinforcing serum ATF3 as a prognostic surrogate of ICH.

## Graphical abstract

Elevated serum ATF3 levels following intracerebral hemorrhage are closely correlated with disease severity and effectively predict early neurological deterioration, stroke‐associated pneumonia and poor prognosis, reinforcing serum ATF3 as a prognostic surrogate of intracerebral hemorrhage.

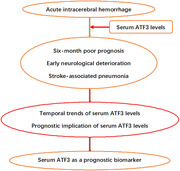

## 1 Introduction

Spontaneous ICH belongs to a serious cerebrovascular accident that gravely endangers human health and poses a massive threat to the economy and society (Puy et al. 2024). Virtually, primary ICH is attributed to non‐traumatic cerebrovascular rupture, which is pathologically ascribed to cerebrovascular atherosclerosis or amyloidosis (Greenberg et al. 2022). Mechanistically, hematoma directly gives rise to physical deformation of neurons and glia, and concurrently evokes a plethora of cascading molecular events with intricate interplay, covering inflammatory reactions, oxidative responses, cellular apoptosis, and more, thereby damaging blood–brain barrier permeability, aggravating brain edema, incurring neuron demise, ultimately leading to neurological functional deficits, and even bringing about individual death (Lee 2025; Magid‐Bernstein et al. 2022). The NIHSS and hematoma volume are the two frequently‐used metrics of severity appraisal, and the mRS is the grading system for properly determining neurological conditions of patients with ICH (Puy et al. 2024; Greenberg et al. 2022; Lee 2025). ICH patients are susceptible to END and SAP, both of which easily put patients at risk of poor prognosis (Zhu et al. 2024; Wang et al. 2023). As a consequence, it is indispensable to timely and accurately anticipate bad neurological state, END, and SAP during the clinical practice of ICH management. On account of the easy availability of blood specimens, blood biochemical indicators, such as cellular prion protein, α‐melanocyte‐stimulating hormone, vascular endothelial growth factor, glial fibrillary astrocyte protein, S100 calcium binding protein B and so forth, have gained close attention during recent decades due to their good clinical practicability in severity appraisal and outcome prediction of ICH (Wu et al. 2022; Hu et al. 2023; Troiani et al. 2021).

ATF3 is a member of the ATF/ cAMP response element binding protein family and structurally harbors a basic leucine zipper (Zhou et al. 2018). ATF3 has a low expression level in quiescent cells, while its expression can be rapidly raised under stress and pathological circumstances, collectively shedding light on the role of ATF3 as an early phase‐response protein (Hai et al. 1999). Of note, ATF3 is clearly admitted as a “hub” of the cellular adaptive‐response network, giving supportive evidence to the conception that ATF3 may functionally act as a compensatory factor (Lin and Cheng 2018; Hai et al. 2010). In the central nervous system, ATF3 expression by microglia and neurons was obviously enhanced in reaction to acute brain injury (Hunt et al. 2012). Compelling experimental data of acute brain injury have alluded to the hypothesis that ATF3 may hold brain protective functions via modulating inflammatory responses (Li et al. 2023). Noteworthily, blood ATF3 levels were substantially elevated in both mice and humans with spinal cord injury or acute ischemic stroke, and blood ATF3 levels were positively related to neurological impairments of patients with ischemic stroke as well (Pan et al. 2024). Collectively, ATF3 may emerge as a potential biomarker of acute brain injury. Currently, serum ATF3 levels are quantified, with the intent to unravel their temporal kinetics and further to unveil its predictive values on SAP, END, and poor neurological function in human ICH.

## 2 Materials and Methods

### 2.1 Participants and Ethical Consent

**FIGURE 1:**
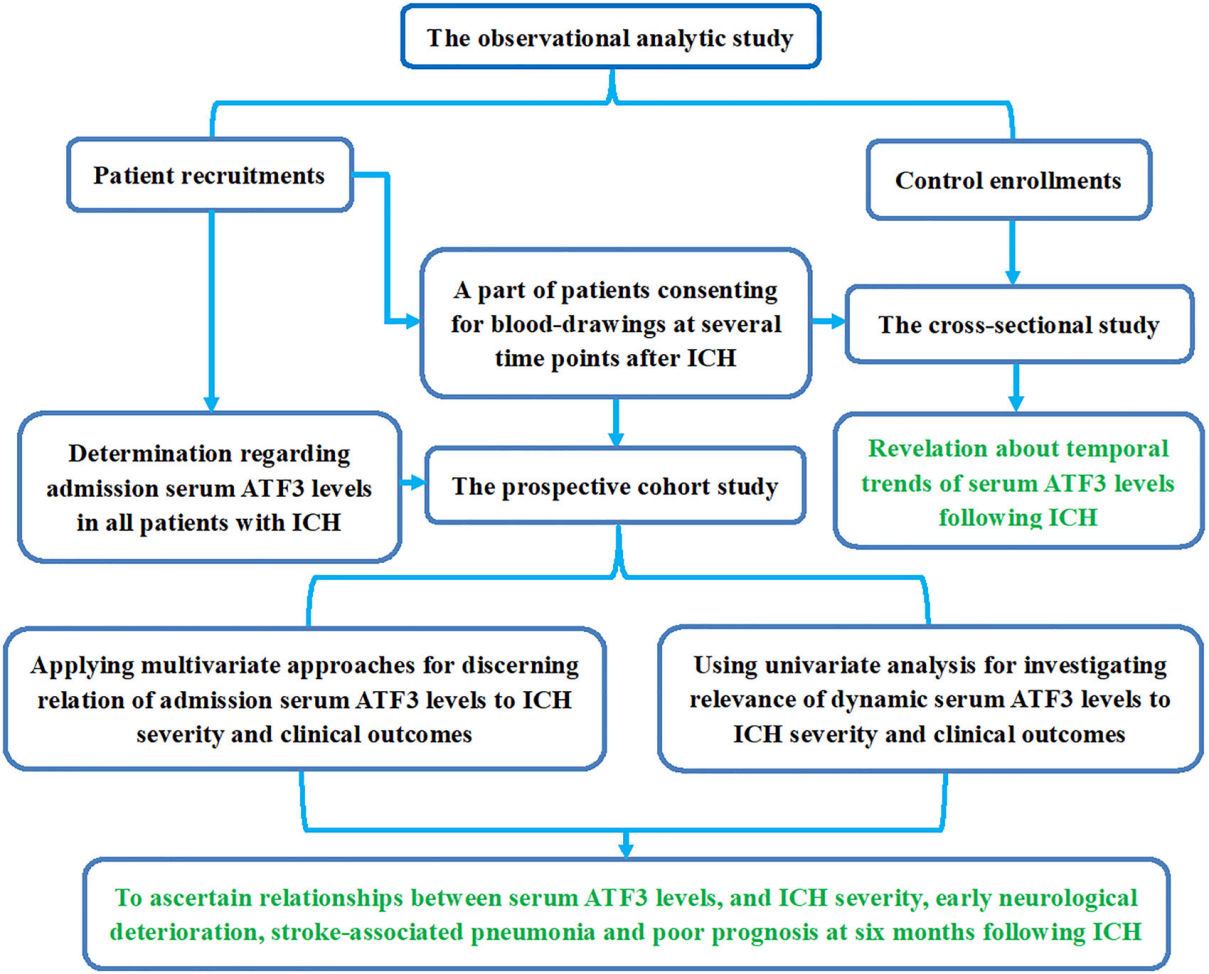
Study design and participant enrollments. In this diagrammatic sketch, the observational analytical study was allocated to two sub‐studies, namely, the prospective cohort study and cross‐sectional study. Patients and controls were recruited to determine the evolutionary trajectory of serum‐based activating transcription factor three levels after acute intracerebral hemorrhage and provide further insight into its prognostic potential. AFT3 stands for ATF3; ICH, intracerebral hemorrhage.

This two‐center observational analytical study was implemented at the Seventh People's Hospital of Shanghai University of Traditional Chinese Medicine (Shanghai, China) and Shanghai Baoshan Luodian Hospital (Shanghai, China) between January 2021 and January 2024. As depicted in Figure 1, this study was divided into two parts, that is, the cross‐sectional sub‐study and prospective cohort sub‐study. In the former, patients who voluntarily provided blood specimens from admission up to post‐ICH day 14, and controls, were assigned to explore the evolutional trajectory of serum ATF3 levels following ICH. In the latter, all patients were arranged for observing SAP, END, and poor prognosis after ICH, and admission serum ATF3 levels were investigated with respect to their relevance to illness severity and clinical outcomes in this entity of ICH. Consecutive patient enrollment was performed. The inclusion criteria were as follows: (1) supratentorial intraparenchymal bleeding on computed tomography (CT) imaging; (2) age of at least 18 years; (3) transferal to hospital within post‐ICH 24 h; (4) first‐in‐life stroke; (5) intracerebral bleeding of non‐secondary causes; (6) non‐surgical treatment of hematoma; and (7) pre‐stroke mRS score of 0. Exclusion criteria included: (1) presence of certain neurological sicknesses, e.g., ischemia or hemorrhagic stroke, intracranial space‐occupying lesions, moderate‐severe craniocerebral trauma, infections, and multiple sclerosis; and (2) severe illnesses in other aspects or peculiar conditions, for example, malignancies, other organ dysfunctions, use of immunosuppressive medications, pre‐stroke respiratory system infections, indications for mechanical ventilation, missed visits, inadequate clinical materials, denying for participation, and unqualified blood samples. Controls were not diseased of some chronic illnesses, encompassing hypertension, diabetes mellitus, dyslipidemia, and others, and concurrently possessed normal results in physiological examinations and some routine tests, covering blood leukocyte counts, blood glucose levels, chest radiography, and so forth. This study was conducted in compliance with local and institutional ethical and legal regulations, as well as the Declaration of Helsinki and its later amends. The study protocol was approved by the Ethics Committees of the Seventh People's Hospital of Shanghai University of Traditional Chinese Medicine (Shanghai, China) and Shanghai Baoshan Luodian Hospital (Shanghai, China) (joint approval number: 2024‐7th‐HIRB‐169). Patients’ statutory representatives and controls themselves were informed of the study contents and signed informed consent forms autonomously.

### 2.2 Data Gatherings

Demographical data (age and sex), adverse lifestyle habits (cigarette smoking and alcohol consumption), common chronic diseases (hypertension, diabetes mellitus, dyslipidemia, chronic obstructive pulmonary disease, ischemic heart disease, and hyperuricemia), use of medications (statins, anticoagulants and antiplatelet agents) and vital signs (systolic arterial blood pressure and diastolic arterial blood pressure) were inquired or measured and then recorded. Interval since stroke onset up to admission or blood sampling was registered. Admission neurological deficits were assessed via the NIHSS (Kwah and Diong 2014). Patients were carefully observed about clinical symptoms so as to identify vomiting occurrence. Dysphagia was diagnosed by performing the swallowing test. The first head CT imaging was closely checked so as to make clear whether intraparenchymal hematoma was expanded into intraventricular or subarachnoid space. Hematoma types were divided into superficial and deep ones. Hematoma size was formulated using the equation: 0.5 × a × b × c (Kothari et al. 1996). END was deemed as an enhancement of four points or greater in NIHSS score or death within twenty‐four hours following admission into hospital (Law et al. 2021). On the basis of the diagnostic criteria set up by a consensus group, SAP was referred to as the acute onset of lower airway infections within the first seven days subsequent to ICH (Smith et al. 2015). The mRS scores varies from zero to six; higher mRS scores were, worse neurological deficits were; and the score at six point means death. Patients were followed up until six months following ICH for assessing their neurological status by employing mRS, and the scores from 3 to 6 signified poor prognosis (Coleman et al. 2024).

### 2.3 Immune Analysis

All patients supplied admission venous blood samples, and a portion of patients voluntarily continued to provide blood specimens at serial time points, that is, days 1, 3, 5, 7, 10, and 14 following ICH. As for the controls, blood drawings were completed at their recruitment into the study. The antecubital venipuncture technique was utilized for aspirating venous blood, the collected blood specimens were promptly laid inside gel‐containing tubes, and then the samples got centrifugation at 2000 × g for ten min to isolate cells and debris. The supernatants were sucked, with the swift transferal to Eppendorf tubes for preservation at below 80°C condition of the freezer up to later detection. In order not to make protein components decompose, storage duration of blood samples at low‐temperature environment did not transcend three months. For every measurement, the whole batch of serum specimens were melted simultaneously. Enzyme‐linked immunosorbent assay kit for gauging serum ATF3 levels was offered by Aviva Systems Biology (catalogue number: OKDD01469). The assay range was 15.6 to 1000 pg/ml, the minimum detectable dose was typically 7.36 pg/ml, and both intra‐ and inter‐assay coefficients of variation were below 10%. As per the kit specifications, dual measurements were done of each sample by the same proficient technician of inaccessibility to clinical contents. By transforming the two measurements into mean values, later statistical analyses were fulfilled.

### 2.4 Statistical Analysis

The four software packages (SPSS 23.0 [SPSS Inc., Chicago, IL, USA], R 3.5.1 [www.r‐project.org], GraphPad Prism 7.01 [GraphPad Software, Inc., San Diego, California, USA], and MedCalc 20 [MedCalc Software, Ltd., Ostend, Belgium]) were operated to analyze data and compile graphs. Variables were of two types, that is, categorical and numerical. The former was divided into nominal and ordinal as well and was presented as count (proportion), and the latter was classified into continuous and discrete and was reported as mean (standard deviation) or median (lower‐upper quartiles) in accordance with normality by the Kolmogorov–Smirnov test. The chi‐square test and Fisher's exact test were performed for two‐group or multiple‐group comparisons of categorical variables; the independent‐sample Student's t‐test, Mann–Whitney U test, Kruskal–Wallis test, and one‐way analysis of variance (ANOVA) test, for those of numerical variables. Bivariable correlations were assessed using Spearman's test. Here, three kinds of multivariate models, that is, linear regression approach, binary logistic regression analysis, and ordinal regression method, were established, in which continuous serum ATF3 levels, discrete mRS scores, ordinal mRS scores, binary mRS scores (poor prognosis versus good prognosis), END and SAP were dependent variables. All variables of significant relevance through univariate analyses were forced into the respective multivariate models to initiate analyses. The pertinences are reported as beta (β) or odds ratio (OR) values as applicable, with corresponding 95% confidence intervals (CIs). Alternatively, the Hosmer‐Lemeshow goodness‐of‐fit test was made for a generalized linear model fitted to binary responses, the restricted cubic spline (RCS) was graphed for linearity assessment (Desquilbet and Mariotti 2010), the E‐value was estimated for sensitivity analysis (Vale et al. 2021), as well as subgroup analysis, which was visualized by the forest plot, was performed, and accompanied by the likelihood ratio test for interactional appraisal. The ROC curve was plotted as well, followed by calculation of the area under curve (AUC) for discrimination efficiency evaluation. The variance inflation factor (VIF) value was computed for judging multicollinearity in regression models (Kim 2019). A type 1 error value (alpha) of 0.05, a test power (1‐β) of 0.95, and an effect size of 0.8 were selected to estimate sample size for the sake of comparing serum ATF3 levels statistically. Verification of the accuracy as to sample size estimation was finished by operating the priori power analysis within G*Power 3.1.9.4 software (Heinrich‐Heine Universität Düsseldorf, Germany). For all analyses, differences were designated as statistical significance when the two‐tailed *p*‐value was less than 0.05.

## 3 Results

### 3.1 Study Subjects

Initially, a collective of 302 patients were evaluated based on the study inclusion criteria. Afterwards, a total of 236 patients were given the final clinical investigation, following the removal of 66 cases from the study cohort on the basis of the exclusion criteria. Among them, 114 patients volunteered to allow for serial blood drawings following ICH. Aggregately, 114 controls were added to the current study. As displayed in Table 1, no substantial differences were statistically demonstrable in terms of age, sex, tobacco smoking, and alcohol drinking among all patients, those consenting for serial sampling and controls (all *p* > 0.05), while other variables, such as chronic diseases, medications, radiological appearances, and so on, were non‐statistically significantly different between all patients and those consenting for serial sampling (all *p* > 0.05).

**TABLE 1:** Baseline features between controls and patients with acute intracerebral hemorrhage.

Variables	All patients	Specific patients	Controls	*p* values
Number	236	114	114	—
Age (years)	61.4 ± 10.4	62.2 ± 10.6	59.9 ± 10.9	0.252
Gender (male/female)	137/99	64/50	65/49	0.941
Cigarette smoking	80 (33.9%)	37 (32.5%)	35 (30.7%)	0.834
Alcohol drinking	83 (35.2%)	40 (35.1%)	36 (31.6%)	0.785
Hypertension	153 (64.8%)	79 (69.3%)	—	0.407
Diabetes mellitus	47 (19.9%)	25 (21.9%)	—	0.662
Dyslipidemia	81 (34.3%)	41 (36.0%)	—	0.762
COPD	10 (4.2%)	7 (6.1%)	—	0.438
Ischemic heart ischemia	17 (7.2%)	12 (10.5%)	—	0.291
Hyperuricemia	28 (11.9%)	14 (12.3%)	—	0.911
Previous statin use	58 (24.6%)	28 (24.6%)	—	0.998
Previous anticoagulant use	14 (5.9%)	5 (4.4%)	—	0.550
Previous antiplatelet use	30 (12.7%)	15 (13.2%)	—	0.907
Admission time (h)	8.1 (4.5–13.5)	7.5 (3.5–13.5)	—	0.343
Sampling time (h)	8.8 (5.5–14.5)	8.5 (4.5–14.5)	—	0.499
Dysphasia	52 (22.0%)	25 (21.9%)	—	0.982
Vomiting	63 (26.7%)	32 (28.1%)	—	0.786
Systolic arterial pressure (mmHg)	142.3 ± 27.8	141.5 ± 28.5	—	0.804
Diastolic arterial pressure (mmHg)	89.5 ± 12.6	88.9 ± 12.6	—	0.728
Hemorrhagic locations (superficial/deep)	62/174	30/84	—	0.993
Intraventricular expansion of hematoma	37 (15.7%)	17 (14.9%)	—	0.853
Subarachnoidal expansion of hematoma	8 (3.4%)	4 (3.5%)	—	0.954
NIHSS scores	9 (6–14)	9.5 (6–14)	—	0.798
Hematoma volume (ml)	14 (11–23)	15 (10–24)	—	0.969
Blood leucocyte count (× 10^9^/l)	6.5 (4.7–9.4)	6.8 (4.7–10.0)	—	0.936
Blood glucose levels (mmol/l)	9.5 (7.7–12.0)	9.8 (7.9–12.1)	—	0.583
Six‐month mRS (continuous type)	2 (1–4)	2 (1–4)	—	0.826
Six‐month mRS (categorical type)	—	—	—	0.830
0	18	9	—	—
1	43	20	—	—
2	69	32	—	—
3	36	17	—	—
4	27	15	—	—
5	31	15	—	—
6	12	6	—	—
Six‐month poor prognosis	106 (44.9%)	53 (46.5%)	—	0.781
Early neurological deterioration	77 (32.6%)	36 (31.6%)	—	0.844
Stroke‐associated pneumonia	63 (26.7%)	32 (28.1%)	—	0.786

*Notes*: Variables were summarized as count (percentage), mean ± standard deviation or median (upper‐lower quartiles). As applicable, the Chi‐square test, Fisher exact test, Student's 𝑡‐test, Mann‐Whitney test, one‐way ANOVA or Kruskal‐Wallis H test was in use for comparing data. Specific patients refer to those agreeing with blood‐collection at numerous time points.

Abbreviations: NIHSS, indicates National Institutes of Health Stroke Scale; mRS, modified Rankin Scale; COPD, chronic obstructive pulmonary disease.

### 3.2 Temporal Trends of Serum ATF3 Levels and Their Pertinence to ICH Severity

**FIGURE 2:**
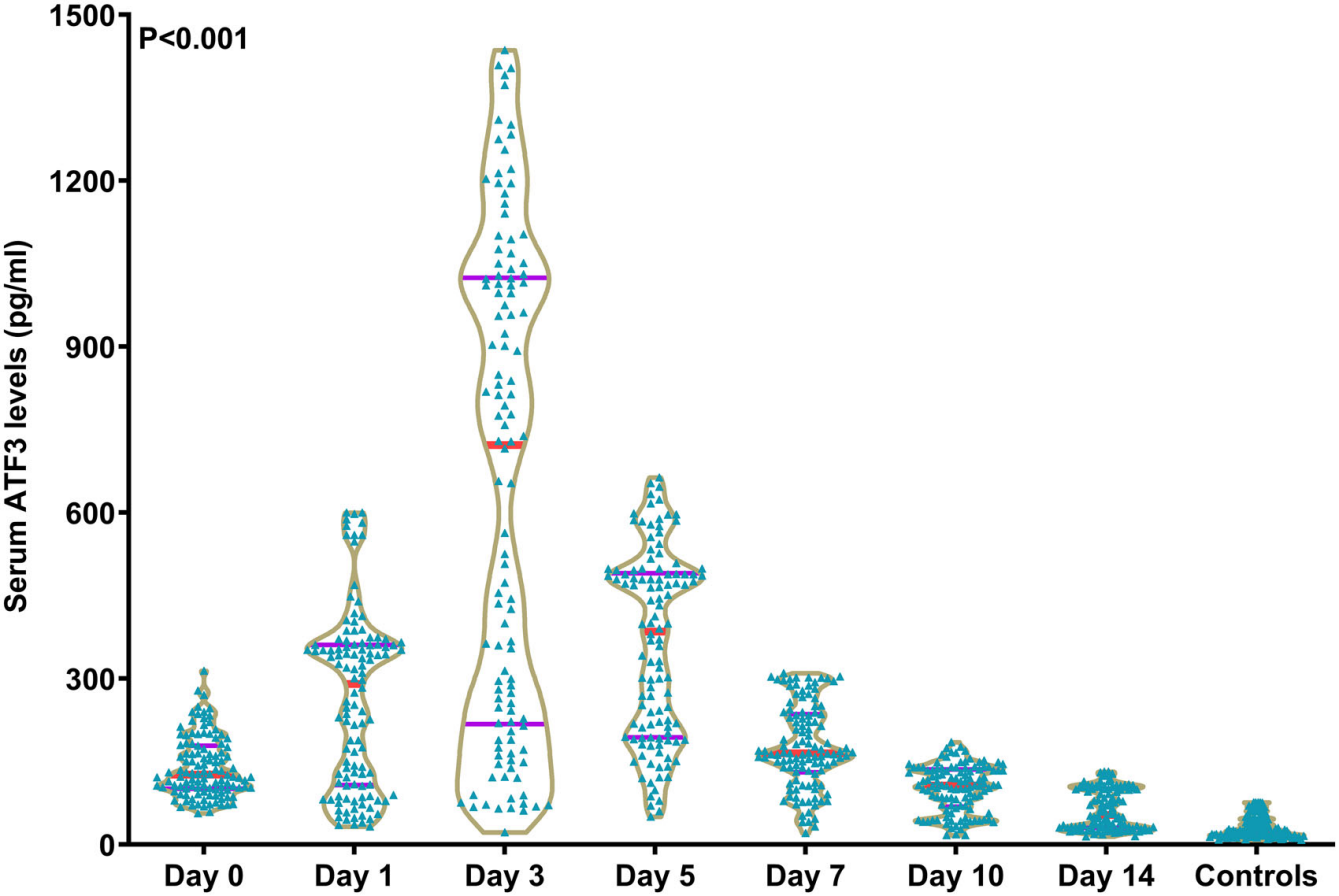
Longitudinal variation of serum activating transcription factor three levels following acute intracerebral hemorrhage. In this violin plot, patients with acute intracerebral hemorrhage had significantly higher serum ATF3 levels from admission up to day 14 following stroke than controls, along with a peak value on day 3 (*p* < 0.001). ATF3 is indicative of activating transcription factor 3.

Serum ATF3 levels exhibited a rapid rise at admission of patients consenting for serial sampling, as compared to controls; the levels were thereafter continuously heightened at day 1 post‐ICH, finally reached their highest point at day 3, were subsequently gradually reduced up to day 14 following ICH; and the levels were significantly higher during the 14‐day period in patients allowing for serial sampling than in controls (*p* < 0.001; Figure 2). Among patients permitting dynamic sampling, both NIHSS scores and hematoma volume were profoundly positively relevant to serum ATF3 levels at serial time points post‐ICH (all P<0.001; Supplementary Table 1). For all patients, serum ATF3 levels at admission were tightly related to both NIHSS scores and hematoma volume (both *p* < 0.001; Supplementary Figure 1 and Supplementary Figure 2). As outlined in Table 2, besides NIHSS score and hematoma volume, other variables of intimate pertinence to admission serum ATF3 levels in all patients included dysphagia, vomiting, intraventricular enlargement of the hematoma, and blood glucose levels (all *p* < 0.05). With the addition of the above‐mentioned six factors into the multivariate linear model, NIHSS scores (β = 4.783; 95% CI: 2.604–6.962; VIF = 3.020; *p* = 0.001) and hematoma volume (β = 1.967; 95% CI = 0.571–3.363; VIF = 3.115; *p* = 0.006) were independently correlated with admission serum ATF3 levels of all patients.

**TABLE 2:** Bivariate correlation analysis in all patients with acute intracerebral hemorrhage.

Variables	Serum ATF3 levels		Modified rankin scale scores	
	*ρ*	*p* values	*ρ*	*p* values
Age (years)	0.115	0.078	0.215	0.001
Gender (male/female)	−0.014	0.825	−0.078	0.232
Cigarette smoking	0.122	0.061	0.025	0.705
Alcohol drinking	0.061	0.353	−0.035	0.590
Hypertension	−0.025	0.703	−0.030	0.647
Diabetes mellitus	0.105	0.109	0.117	0.073
Dyslipidemia	−0.012	0.852	0.031	0.639
Chronic obstructive pulmonary disease	−0.003	0.959	0.100	0.124
Ischemic heart ischemia	−0.016	0.807	0.008	0.897
Hyperuricemia	−0.059	0.365	0.053	0.414
Previous statin use	0.026	0.696	0.113	0.083
Previous anticoagulant use	0.002	0.974	−0.015	0.821
Previous antiplatelet use	0.027	0.675	0.016	0.809
Admission time (h)	0.041	0.536	−0.073	0.266
Sampling time (h)	0.039	0.548	−0.060	0.358
Dysphasia	0.252	< 0.001	0.345	< 0.001
Vomiting	0.181	0.005	0.213	0.001
Systolic arterial pressure (mmHg)	−0.008	0.897	−0.022	0.733
Diastolic arterial pressure (mmHg)	0.004	0.956	−0.052	0.425
Hemorrhagic locations (superficial/deep)	0.002	0.970	−0.015	0.822
Intraventricular expansion of hematoma	0.209	0.001	0.254	<0.001
Subarachnoidal expansion of hematoma	0.032	0.623	0.055	0.404
NIHSS scores	0.553	< 0.001	0.593	< 0.001
Hematoma volume (ml)	0.554	< 0.001	0.607	< 0.001
Blood leucocyte count (× 10^9^/l)	0.065	0.323	0.141	0.030
Blood glucose levels (mmol/l)	0.158	0.015	0.177	0.006
Serum ATF3 levels (pg/ml)	—	—	0.601	< 0.001

*Note*: The Spearman test was used. NIHSS indicates National Institutes of Health Stroke Scale; ATF3, activating transcription factor 3.

### 3.3 Serum ATF3 Levels in Connection With Neurological Outcome at Six Months post‐ICH

Six‐month mRS scores of patients consenting for serial sampling were highly correlated with dynamic serum ATF3 levels (all *p* < 0.001; Supplementary Table 1). As illustrated in Supplementary Figure 3, serum‐based ATF3 levels at admission were substantially related to mRS scores at six months after acute ICH in all patients (*p* < 0.001). Admission serum‐based ATF3 levels, along with age, dysphagia, vomiting, intraventricular expansion of the hematoma, NIHSS scores, hematoma volume, blood leukocyte counts, and blood glucose levels, were closely related to the mRS scores of all patients (all *p* < 0.05; Table 2). The entrance of the aforementioned significant variables on univariate analyses into the multifactorial model led to the revelation that admission serum ATF3 levels (β = 0.018; 95% CI = 0.009–0.026; VIF = 1.702; *p* = 0.004), NIHSS scores (β = 0.148; 95% CI = 0.083–0.212; VIF = 3.034; *p* = 0.002), and hematoma volume (β = 0.087; 95% CI = 0.026‐0.148; VIF = 3.291; *p* = 0.008) kept independently related to mRS scores of all patients.

Patients volunteering for serial sampling were divided into seven subgroups at a basis of mRS, and serum ATF3 levels on the seven time points were significantly raised among subgroups in order of mRS scores from 0 to 6 (all *p* < 0.001; Supplementary Table 2). Supplementary Figure 4 shows that, in all patients, serum ATF3 levels at admission were markedly lowest in subgroup with the score at 1, followed by those with the score from 1 to 5, and were apparently highest in that with the score at 6 (*p* < 0.001). To be continued, age, dysphagia, vomiting, accumulation of bleeding within the intraventricular space, NIHSS scores, hematoma volume, blood glucose levels and admission ATF3 levels displayed substantial differences among subgroups with different mRS scores in all patients (all *p* < 0.05; Table 3). Next, the preceding variables were forced into the ordinal regression model, and it was proved that admission serum ATF3 levels (OR = 1.018; 95% CI = 1.007–1.029; VIF = 1.991; *p* = 0.009), NIHSS scores (OR = 1.165; 95% CI = 1.059–1.284; VIF = 3.229; *p* = 0.002), and hematoma volume (OR = 1.102; 95% CI = 1.018–1.192; VIF = 3.481; *p* = 0.004) remained independently associated with ordinal mRS scores among all patients.

**TABLE 3:** Variables among subgroups across mRS in all patients with acute intracerebral hemorrhage.

Factors	Modified Rankin Scale scores							*p* values
	0	1	2	3	4	5	6	
Age (years)	55 (48–59)	59 (51–70)	60 (53–66)	61 (51–71)	60 (58–70)	68 (53–76)	69 (62–78)	0.018
Gender (male/female)	12/6	26/17	40/29	23/13	15/12	15/16	6/6	0.842
Cigarette smoking	6 (33.3%)	15 (34.9%)	21 (30.4%)	14 (38.9%)	8 (29.6%)	10 (32.3%)	6 (50.0%)	0.876
Alcohol drinking	8 (44.4%)	16 (37.2%)	22 (31.9%)	13 (36.1%)	10 (37.0%)	10 (32.3%)	4 (33.3%)	0.973
Hypertension	10 (55.6%)	29 (67.4%)	50 (72.5%)	21 (58.3%)	14 (51.9%)	22 (71.0%)	7 (58.3%)	0.427
Diabetes mellitus	3 (16.7%)	9 (20.9%)	6 (8.7%)	9 (25.0%)	9 (33.3%)	7 (22.6%)	4 (33.3%)	0.098
Dyslipidemia	4 (22.2%)	12 (27.9%)	29 (42.0%)	13 (36.1%)	11 (40.7%)	7 (22.6%)	5 (41.7%)	0.370
COPD	1 (5.6%)	1 (2.3%)	1 (1.4%)	1 (2.8%)	3 (11.1%)	1 (3.2%)	2 (16.7%)	0.135
Ischemic heart ischemia	1 (5.6%)	2 (4.7%)	6 (8.7%)	5 (13.9%)	1 (3.7%)	0 (0%)	2 (16.7%)	0.266
Hyperuricemia	2 (11.1%)	3 (7.0%)	7 (10.1%)	8 (22.2%)	5 (18.5%)	0 (0%)	3 (25.0%)	0.058
Previous statin use	0 (0%)	8 (18.6%)	19 (27.5%)	13 (36.1%)	9 (33.3%)	6 (19.4%)	3 (25.0%)	0.083
Previous anticoagulant use	2 (11.1%)	1 (2.3%)	5 (7.2%)	3 (8.3%)	1 (3.7%)	1 (3.2%)	1 (8.3%)	0.780
Previous antiplatelet use	2 (11.1%)	3 (7.0%)	13 (18.8%)	4 (11.1%)	2 (7.4%)	4 (12.9%)	2 (16.7%)	0.597
Admission time (h)	6.6 (4.2–13.5)	9.9 (5.7–15.7)	8.5 (4.5–14.5)	8.4 (4.9–11.2)	6.5 (3.2–8.8)	6.7 (4.5–10.5)	11.4 (8.6–15.1)	0.097
Sampling time (h)	7.4 (5.5–14.0)	10.5 (6.8–16.6)	9.5 (5.3–15.5)	9.2 (5.8–12.2)	7.5 (3.7–9.6)	8.0 (5.4–11.7)	12.7 (9.3–15.7)	0.130
Dysphasia	1 (5.6%)	1 (2.3%)	12 (17.4%)	7 (19.4%)	15 (55.6%)	10 (32.3%)	6 (50.0%)	< 0.001
Vomiting	3 (16.7%)	8 (18.6%)	14 (20.3%)	10 (27.8%)	8 (29.6%)	13 (41.9%)	7 (58.3%)	0.032
Systolic arterial pressure (mmHg)	136 (124–176)	129 (119–140)	139 (127–173)	129 (121–140)	134 (128–180)	131 (119–152)	132 (121–145)	0.129
Diastolic arterial pressure (mmHg)	93 (79–107)	86 (80–98)	90 (84–101)	84 (79–92)	87 (83–103)	86 (82–98)	86 (84–94)	0.264
Hemorrhagic locations (superficial/deep)	5/13	12/31	19/50	7/29	7/20	8/23	4/8	0.971
Intraventricular expansion of hematoma	0 (0%)	2 (4.7%)	9 (13.0%)	7 (19.4%)	7 (25.9%)	8 (25.8%)	4 (33.3%)	0.017
Subarachnoidal expansion of hematoma	0 (0%)	1 (2.3%)	3 (4.3%)	1 (2.8%)	0 (0%)	3 (9.7%)	0 (0%)	0.409
NIHSS scores	5 (1–7)	6 (5–8)	9 (7–12)	11 (9–15)	12 (9–14)	14 (12–17)	15 (12–15)	< 0.001
Hematoma volume (ml)	8 (6–10)	10 (8–12)	12 (11–24)	18 (13–24)	19 (15–24)	25 (20–31)	21 (18–23)	< 0.001
Blood leucocyte count (× 10^9^/l)	6.9 (5.1–7.8)	5.9 (4.9–7.1)	6.0 (4.8–8.9)	7.1 (4.2–10)	7.4 (6.0–9.7)	7.0 (4.4–9.5)	10.1 (8.1–12.1)	0.113
Blood glucose levels (mmol/l)	9.5 (7.1–10.5)	8.5 (6.3–10.5)	8.9 (7.7–10.7)	9.9 (7.9–13.8)	9.8 (8.0–14.4)	11.3 (9.1–15.8)	8.5 (3.9–11.5)	0.008
Serum ATF3 levels (pg/ml)	74.9 (68.7–80.9)	123.1 (99.2–131.9)	121.5 (102.5–138.5)	106.3 (96.6–197.8)	148.5 (115.6–185.0)	201.7 (182.0–242.6)	212.0 (181.9–240.0)	< 0.001

*Notes*: Variables were reported as count (percentage) or median (upper‐lower quartiles) as deemed appropriate, with the Chi‐square test or Kruskal‐Wallis H test in use for statistical analysis.

Abbreviations: NIHSS indicates National Institutes of Health Stroke Scale; COPD, chronic obstructive pulmonary disease; ATF3, activating transcription factor 3.

**FIGURE 3:**
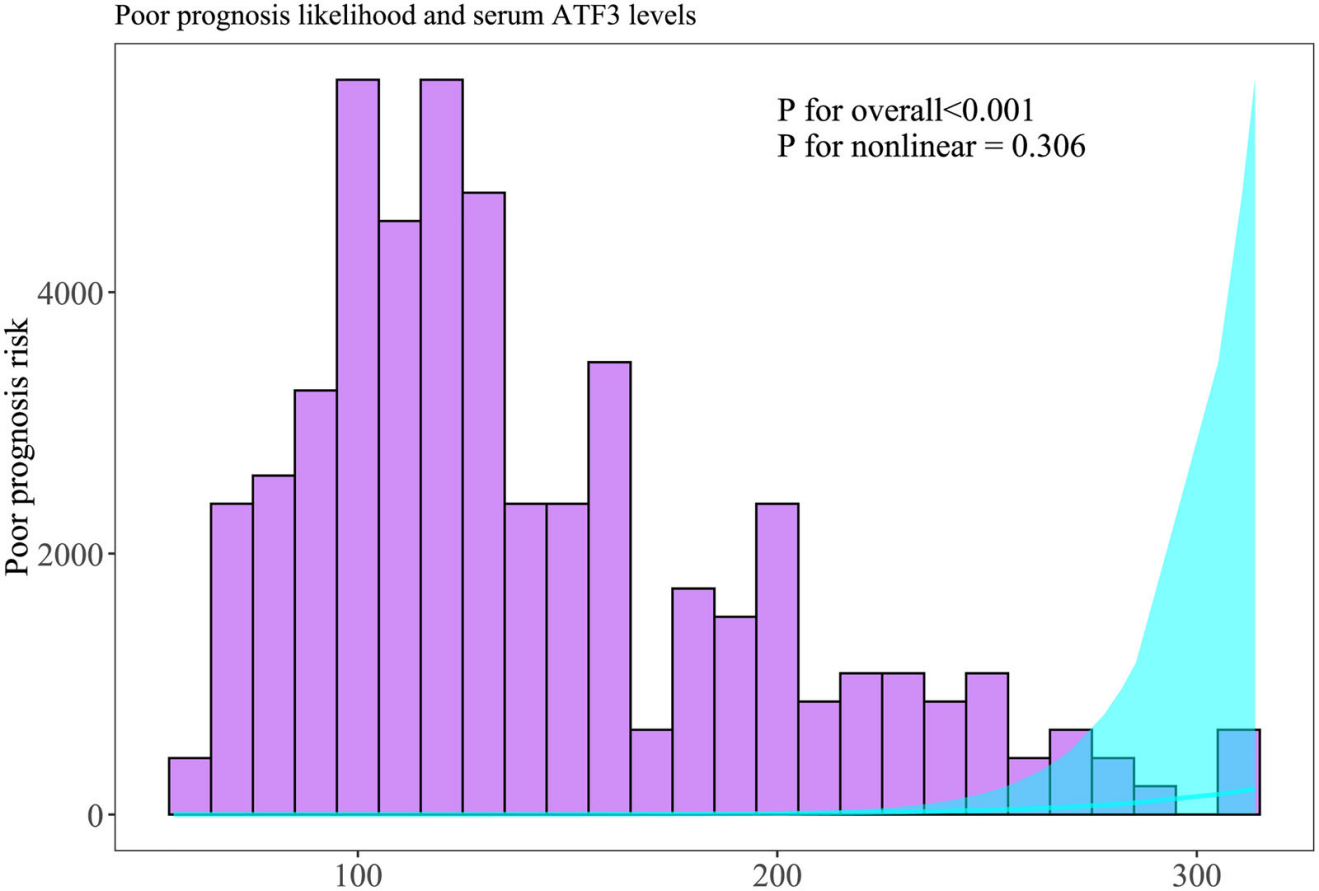
Linearity relationship between serum ATF3 levels at admission and possibility of poor prognosis six months following acute intracerebral hemorrhage. In the framework of restricted cubic spline analysis, serum levels of ATF3 at admission were linearly connected with the chance of poor prognosis in patients encountering acute intracerebral hemorrhage (*p* for nonlinear > 0.05). ATF3 represents activating transcription factor 3.

In Supplementary Table 3, dynamic serum ATF3 levels of patients agreeing with serial sampling were pronouncedly higher in patients presenting with poor prognosis than in those with good prognosis (all *p* < 0.001); and admission serum ATF3 levels were in possession of an AUC, which was comparable to those at other time points (all *p* > 0.05), whereas was higher than that at day 14 (*p* < 0.05). In Supplementary Figure 5, serum ATF3 levels at admission of all patients were profoundly promoted in patients with the development of poor prognosis, as compared to the other remainders (*p* < 0.001). Within the scenario of the RCS appraisal, serum ATF3 levels at admission of all patients were linearly connected to poor prognosis probability (*p* for nonlinearity > 0.05; Figure 3). In the paradigm of the ROC curve analysis, admission serum ATF3 levels comparatively well distinguished the risk of poor prognosis of all patients, as confirmed by the AUC at 0.770, and its threshold value was selected at 155.8 pg/ml by aidance of the Youden method so that poor prognosis was forecasted with the maximum Youden value of 0.538 (Supplementary Figure 6).

In Table 4, subjects with poor prognosis in all patients, in contrast to those without such an experience, tended to have significantly higher age, were markedly more likely to be diseased with diabetes mellitus, were prone to have notably higher proportions of dysphagia, vomiting, and intraventricular hematoma gathering, and were apt to own profoundly higher NIHSS scores, hematoma volume, blood glucose levels, and admission ATF3 levels (all *p* < 0.05). Via integrating the abovementioned highly associated variables into multifactorial model, admission serum ATF3 levels (OR = 1.016; 95% CI:1.008–1.025; VIF = 2.318; *p* = 0.010), NIHSS score (OR = 1.156; 95% CI = 1.011–1.320; VIF = 2.872; *p* = 0.005), and hematoma volume (OR = 1.109; 95% CI = 1.010–1.217; VIF = 2.899; *p* = 0.009) independently predicted poor prognosis of all patients. The binary logistic regression model had satisfactory goodness of fit via the Hosmer‐Lemeshow test (*p* = 0.384).

**TABLE 4:** Factors in relation to poor prognosis after acute intracerebral hemorrhage in all patients.

Components	Poor prognosis	Good prognosis	*p* values
Age (years)	63.3 ± 10.6	60.0 ± 10.1	0.015
Gender (male/female)	59/47	78/52	0.502
Cigarette smoking	38 (35.5%)	42 (32.3%)	0.568
Alcohol drinking	37 (34.9%)	46 (35.4%)	0.939
Hypertension	64 (60.4%)	89 (68.5%)	0.196
Diabetes mellitus	29 (27.4%)	18 (13.8%)	0.010
Dyslipidemia	36 (34.0%)	45 (34.6%)	0.916
Chronic obstructive pulmonary disease	7 (6.6%)	3 (2.3%)	0.103
Ischemic heart ischemia	8 (7.5%)	9 (6.9%)	0.854
Hyperuricemia	16 (15.1%)	12 (9.2%)	0.166
Previous statin use	31 (29.2%)	27 (20.8%)	0.132
Previous anticoagulant use	6 (5.7%)	8 (6.2%)	0.873
Previous antiplatelet use	12 (11.3%)	18 (13.8%)	0.562
Admission time (h)	7.5 (4.0–11.5)	8.5 (4.5–15.2)	0.164
Sampling time (h)	8.5 (5.0–12.5)	9.5 (5.5–15.8)	0.248
Dysphasia	38 (35.8%)	14 (10.8%)	< 0.001
Vomiting	38 (35.8%)	25 (19.2%)	0.004
Systolic arterial pressure (mmHg)	139.5 ± 26.1	144.6 ± 29.0	0.161
Diastolic arterial pressure (mmHg)	88.1 ± 11.5	90.5 ± 13.5	0.151
Hemorrhagic locations (superficial/deep)	26/80	36/94	0.583
Intraventricular expansion of hematoma	26 (24.5%)	11 (8.5%)	0.001
Subarachnoidal expansion of hematoma	4 (3.8%)	4 (3.1%)	0.769
NIHSS scores	13 (9–15)	7 (5–10)	< 0.001
Hematoma volume (ml)	21 (15–25)	11 (8–16)	< 0.001
Blood leucocyte count (× 10^9^/l)	7.5 (4.5–10.1)	6.0 (4.8–8.0)	0.061
Blood glucose levels (mmol/l)	10.0 (8.0–14.3)	8.9 (7.5–10.6)	0.002
Serum ATF3 levels (pg/ml)	175.7 (114.8–216.2)	117.5 (95.5–132.0)	< 0.001

*Notes*: Data were presented as count (percentage), mean ± standard deviation or median (upper‐lower quartiles). Statistical methods included the Chi‐square test, Fisher exact test, Student's 𝑡‐test and Mann‐Whitney test.

Abbreviations: NIHSS, National Institutes of Health Stroke Scale; ATF3, activating transcription factor 3.

**FIGURE 4:**
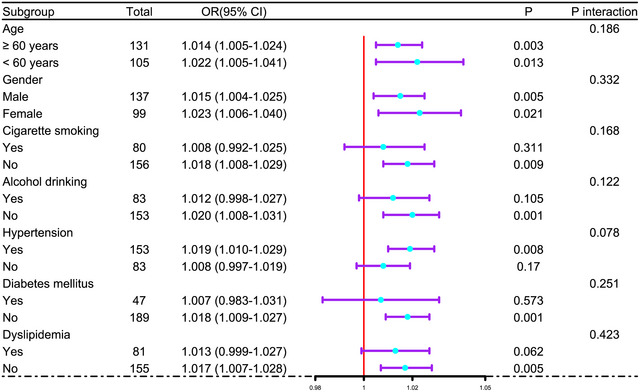
Analysis as to probable moderation effect of conventional parameters on prognosis association following acute intracerebral hemorrhage. In the forest plot, some traditional factors, such as age, sex, tobacco smoking and so on, did not moderate the association of admission serum activating transcription factor three levels in the milieus of subgroup analysis and via the likelihood ratio test (all *p* interaction > 0.05). OR means OR; 95% CI, 95% confidence interval.

**FIGURE 5:**
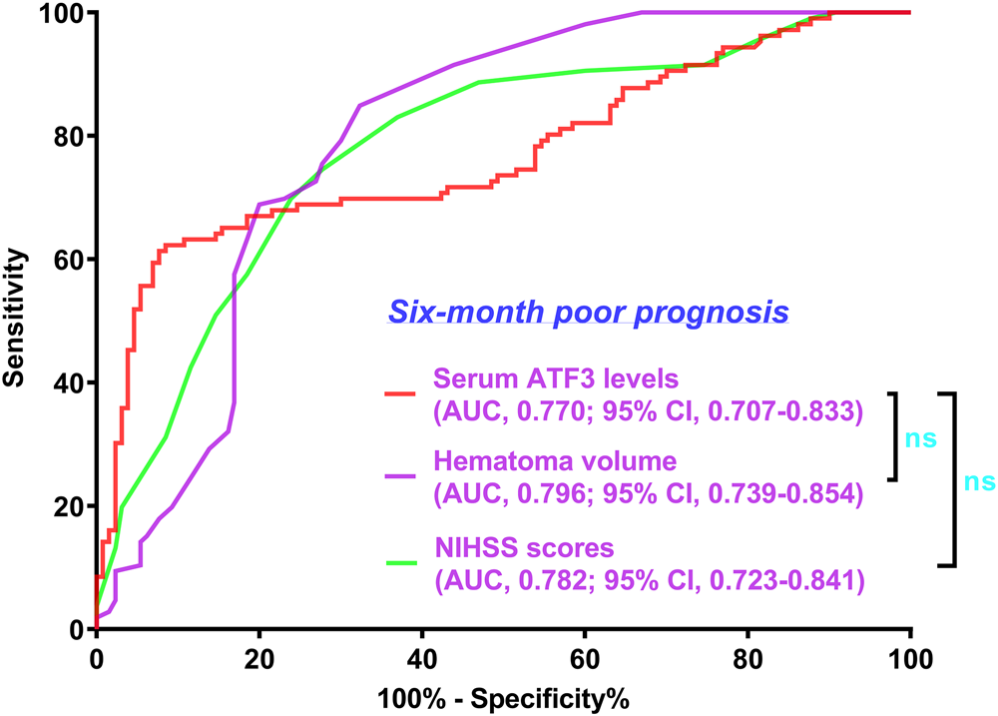
Capability of admission serum activating transcription factor three levels and other indicators for foretelling poor prognosis following acute intracerebral hemorrhage. In the paradigm of ROC curve assessment, admission serum activating transcription factor three levels had analogous predictive effect for poor prognosis, in contrast to the NIHSS scores and hematoma volume in patients with acute intracerebral hemorrhage (both *p* > 0.05). ATF3 signifies activating transcription factor 3; NIHSS, National Institutes of Health Stroke Scale; AUC, area under the curve; 95% CI, 95% confidence interval; ns, non‐significant.

In Supplementary Figure 7, the E‐value for sensitivity assessment was 1.144 (95% CI: 1.098–1.185). Within the framework of subgroup analysis, the association of admission serum ATF3 levels with poor prognosis was negligibly moderated by age, sex, alcohol consumption, and more (all *p* interactions >0.05; Figure 4). In Figure 5, the AUC of admission serum ATF3 levels was analogous to those of NIHSS scores and hematoma volumes for prognosis anticipation in the setting of acute ICH (both *p* > 0.05).

### 3.4 Serum ATF3 Levels in Relation to END Post‐ICH3

Among patients consenting for serial sampling, END subjects, versus non‐END subjects, showed notably heightened serum ATF3 levels at the serial time points (all *p* < 0.01; Supplementary Table 4). Admission serum ATF3 levels, over those at days 1, 3, 5, and 7, were statistically confirmed to be analogously efficient for predicting END (all *p* > 0.05; Supplementary Table 4), and, in contrast to those at days 10 and 14, its ability to predict END was substantially enhanced (both *p* < 0.05; Supplementary Table 4). Likewise, in END individuals, relative to those without END, profoundly increased admission serum ATF3 levels were found even in all patients (*p* < 0.001; Supplementary Figure 8). In help of the RCS analysis, serum ATF3 levels at admission were affirmed to be related to possibility of END in the dose‐response manner among all patients (*p* for nonlinear > 0.05; Figurem 6). Consistently, as exhibited by the AUC greater than 0.75, the END likelihood of all patients was effectively identified by admission serum ATF3 levels, and the criterion value was set at 182.3 pg/ml by applying the Youden approach so as to optimally forecast END, with the maximum Youden index at 0.413 (Supplementary Figure 9).

**FIGURE 6:**
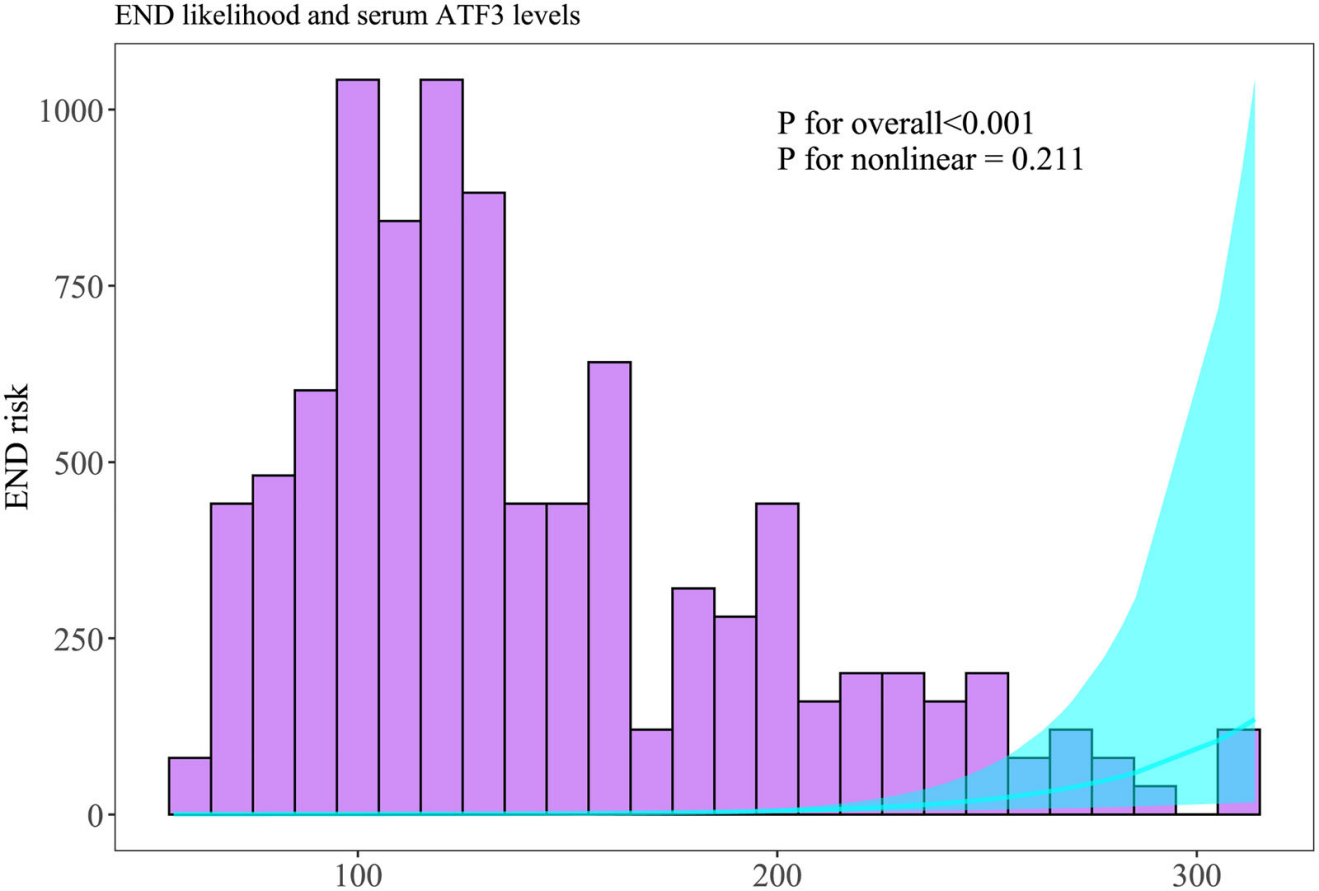
Linearity relationship between serum activating transcription factor three levels at admission and likelihood of END after acute intracerebral hemorrhage. In the context of restricted cubic spline analysis, serum levels of ATF3 at admission were linearly correlated with the risk of END following acute intracerebral hemorrhage (*p* for nonlinear > 0.05). ATF3 denotes activating transcription factor 3; END, early neurological deterioration.

END individuals among all patients, as opposed to the other remainders, were substantially older, had experienced higher chances of previous antiplatelet use, dysphasia, vomiting, and intraventricular extension of hematoma, and possessed markedly elevated NIHSS scores, hematoma volumes, blood glucose levels and admission ATF3 levels (all *p* < 0.05; Table 5). When these variables of significant disparity were put into the binary logistic regression module, it emerged that serum ATF3 levels at admission (OR = 1.014; 95% CI: 1.006–1.023; VIF = 2.207; *p* = 0.015), NIHSS score (OR = 1.272; 95% CI = 1.168–1.383; VIF = 2.621; *p* = 0.010), and hematoma volume (OR = 1.141; 95% CI = 1.092–1.192; VIF = 2.788; *p* = 0.011) were independently associated with END appearance of all patients. The binary logistic regression model showed acceptable goodness of fit through the Hosmer‐Lemeshow test (*p* = 0.401).

**TABLE 5:** Factors in relation to early neurological deterioration after acute intracerebral hemorrhage in all patients.

Components	Early neurological deterioration		*p* values
	Presence	Absence	
Age (years)	63.7 ± 9.8	60.3 ± 10.6	0.020
Gender (male/female)	48/29	89/70	0.353
Cigarette smoking	29 (37.7%)	51 (32.1%)	0.395
Alcohol drinking	33 (42.9%)	50 (31.4%)	0.085
Hypertension	52 (67.5%)	101 (63.5%)	0.545
Diabetes mellitus	17 (22.1%)	30 (18.9%)	0.563
Dyslipidemia	25 (32.5%)	56 (35.2%)	0.676
COPD	1 (1.3%)	9 (5.7%)	0.173
Ischemic heart ischemia	3 (3.9%)	14 (8.8%)	0.171
Hyperuricemia	5 (6.5%)	23 (14.5%)	0.076
Previous statin use	17 (22.1%)	41 (25.8%)	0.535
Previous anticoagulant use	6 (7.8%)	8 (5.0%)	0.400
Previous antiplatelet use	15 (19.5%)	15 (9.4%)	0.030
Admission time (h)	8.5 (5.0–13.9)	7.5 (4.0–13.3)	0.080
Sampling time (h)	9.5 (6.3–14.7)	8.5 (5.0–14.2)	0.117
Dysphasia	24 (31.2%)	18 (17.6%)	0.018
Vomiting	29 (37.7%)	34 (21.4%)	0.008
Systolic arterial pressure (mmHg)	144.9 ± 28.6	141.0 ± 27.4	0.321
Diastolic arterial pressure (mmHg)	91.0 ± 13.6	88.7 ± 12.1	0.205
Hemorrhagic locations (superficial/deep)	18/59	44/115	0.482
Intraventricular expansion of hematoma	25 (32.5%)	12 (7.5%)	< 0.001
Subarachnoidal expansion of hematoma	3 (3.9%)	5 (3.1%)	0.765
NIHSS scores	14 (11–15)	8 (6–10)	< 0.001
Hematoma volume (ml)	24 (17–28)	12 (10–19)	< 0.001
Blood leucocyte count (× 10^9^/l)	7.8 (4.6–10.2)	6.2 (4.8–8.6)	0.088
Blood glucose levels (mmol/l)	10.5 (8.1–13.4)	9.3 (7.3–10.8)	0.028
Serum ATF3 levels (pg/ml)	182.8 (124.1–233.0)	119.3 (96.7–149.3)	< 0.001

*Notes*: Data were presented as count (percentage), mean ± standard deviation or median (upper‐lower quartiles) as considered suitable. The Chi‐square test, Fisher exact test, Student's 𝑡‐test or Mann‐Whitney test was in use for intergroup comparison.

Abbreviations: NIHSS National Institutes of Health Stroke Scale; COPD, chronic obstructive pulmonary disease; ATF3, activating transcription factor 3.

**FIGURE 7:**
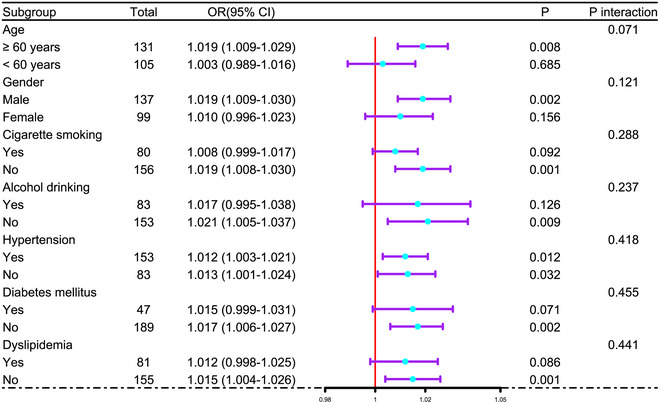
Assessment of moderation effect of conventional parameters on END association subsequent to acute intracerebral hemorrhage. As outlined in the forest graph, age, sex, tobacco smoking, and others displayed non‐interactional properties with admission serum ATF3 levels in terms of association with END based on subgroup analysis and the likelihood ratio test (all *p* interaction > 0.05). OR means odds ratio; 95% CI, 95% confidence interval.

**FIGURE 8:**
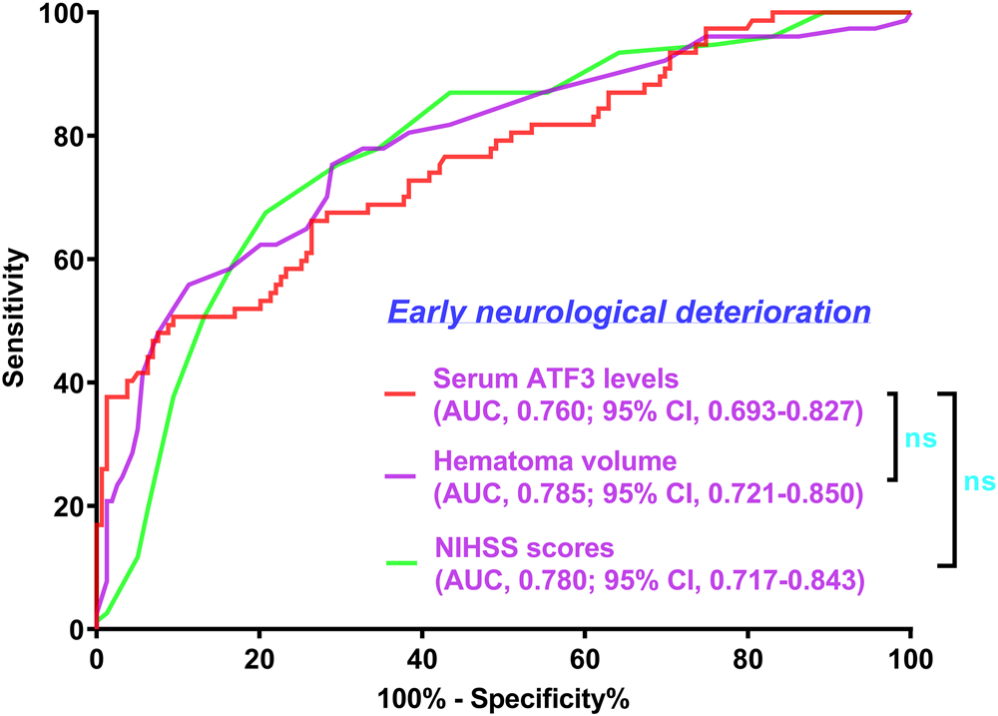
Capability of serum activating transcription factor three levels at admission and other indicators for prognosticating END subsequent to acute intracerebral hemorrhage. In the scenario of ROC curve analysis, admission serum activating transcription factor three levels had the similar predictive ability for END following acute intracerebral hemorrhage, as compared to the NIHSS scores and hematoma volume in patients diagnosed with acute intracerebral hemorrhage (both *p* > 0.05). ATF3 indicates activating transcription factor 3; NIHSS, National Institutes of Health Stroke Scale; AUC, area under the curve; 95% CI, 95% confidence interval; ns, non‐significant.

As depicted in Supplementary Figure 10, the E‐value was 1.133 (95% CI: 1.084–1.176) for sensitivity assessment. In the milieu of subgroup analysis, no substantial interactions were revealed between admission serum ATF3 levels and certain factors, for example, age, sex, smoking, etc. (all *p* > 0.05; Figure 7). Figure 8 shows that the capability of admission serum ATF3 levels to foretell END resembled those of NIHSS scores and hematoma volume (both *p* > 0.05).

### 3.5 Relevance of Serum ATF3 Levels to SAP Following ICH

**FIGURE 9:**
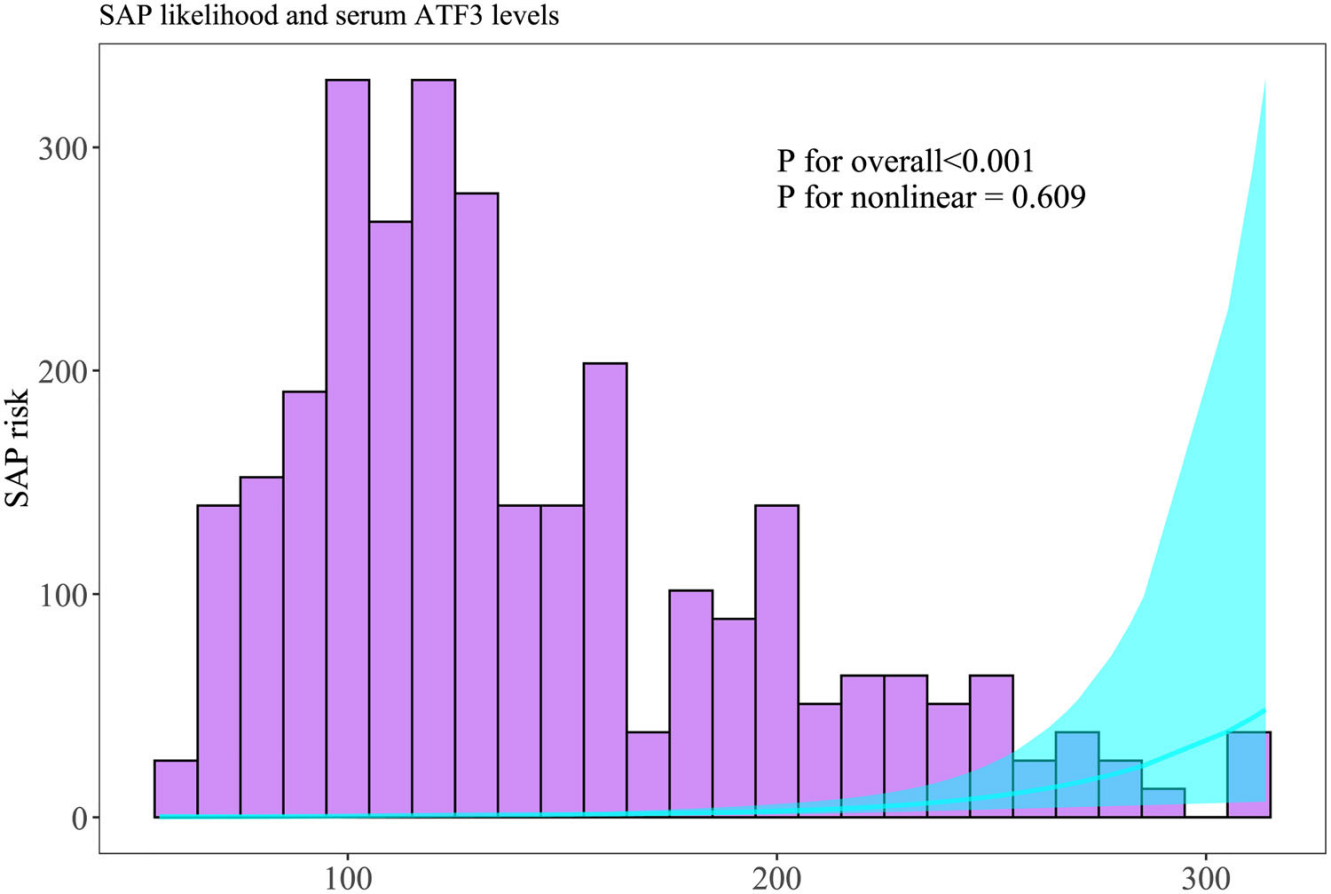
Linear relation of serum activating transcription factor three levels at admission to likelihood of SAP subsequent to acute intracerebral hemorrhage. In accordance with the restricted cubic spline analysis, admission serum activating transcription factor three levels were linearly related to the likelihood of SAP after acute intracerebral hemorrhage (*p* for nonlinear > 0.05). ATF3 indicates activating transcription factor 3; SAP, stroke‐associated pneumonia.

Among patients allowing for serial sampling, serum ATF3 levels at all time points were markedly higher in cases who experienced SAP than in those who not (all *p* < 0.01; Supplementary Table 5). Alternatively, the SAP predictive ability of serum ATF3 levels at admission in the aspect of AUC was equivalent to those at the remaining time points (all *p* > 0.05; Supplementary Table 5). Moreover, among all patients, there was a significant escalation of admission serum ATF3 levels in patients who experienced SAP, as compared to those who not (*p* < 0.001; Supplementary Figure 11). Under the RCS, serum‐based ATF3 levels at admission were linearly correlated with the possibility of SAP in all patients (*p* for nonlinearity > 0.05; Figure 9). Admission serum ATF3 levels owned AUCs above 0.75, and therefore effectively forecasted SAP emergence of all patients, and the maximum Youden value was set at 0.415 for efficaciously distinguishing patients with the risk of SAP (Supplementary Figure 12).

As for all patients, SAP cases, relative to non‐SAP cases, were pronouncedly older; had significantly higher proportions of dysphasia, vomiting, and intraventricular extension of hematoma; and were in possession of markedly higher NIHSS scores, hematoma volumes, blood glucose levels, white blood cell counts, and admission ATF3 levels (all *p* < 0.05; Table 6). Through incorporating these significantly distinct parameters into the multifactorial model, admission serum ATF3 levels (OR = 1.010; 95% CI = 1.002–1.017; VIF = 3.211; *p* = 0.038), NIHSS scores (OR = 1.148; 95% CI = 1.008–1.307; VIF = 3.008; *p* = 0.012), and hematoma volumes (OR = 1.116; 95% CI = 1.068–1.166; VIF = 2.155; *p* = 0.019) appeared as the three independent predictors of SAP among all patients. The binary logistic regression model exhibited rational goodness of fit by using the Hosmer‐Lemeshow test (*p* = 0.298).

**TABLE 6:** Factors in relation to stroke‐associated pneumonia after acute intracerebral hemorrhage in all patients.

Components	Stroke‐associated pneumonia		*p* values
	Yes	No	
Age (years)	64.8±9.3	60.2 ± 10.6	0.002
Gender (male/female)	38/25	99/74	0.670
Cigarette smoking	25 (39.7%)	55 (31.8%)	0.257
Alcohol drinking	27 (42.9%)	56 (32.4%)	0.136
Hypertension	41 (65.1%)	112 (64.7%)	0.961
Diabetes mellitus	14 (22.2%)	33 (19.1%)	0.592
Dyslipidemia	21 (33.3%)	60 (34.7%)	0.847
COPD	2 (3.2%)	8 (4.6%)	0.625
Ischemic heart ischemia	4 (6.3%)	13 (7.5%)	0.759
Hyperuricemia	5 (7.9%)	23 (13.3%)	0.260
Previous statin use	13 (20.6%)	45 (26.0%)	0.396
Previous anticoagulant use	4 (6.3%)	10 (5.8%)	0.870
Previous antiplatelet use	12 (19.0%)	18 (10.4%)	0.078
Admission time (h)	8.5 (4.7–13.5)	8.0 (4.4–13.5)	0.737
Sampling time (h)	9.0 (5.5–14.3)	8.5 (5.5–14.5)	0.748
Dysphasia	24 (38.1%)	28 (16.2%)	< 0.001
Vomiting	26 (41.3%)	37 (21.4%)	0.002
Systolic arterial pressure (mmHg)	141.3 ± 26.2	142.7 ± 28.5	0.736
Diastolic arterial pressure (mmHg)	89.6 ± 12.4	89.4 ± 12.7	0.919
Hemorrhagic locations (superficial/deep)	12/51	50/123	0.128
Intraventricular expansion of hematoma	23 (36.5%)	14 (8.1%)	< 0.001
Subarachnoidal expansion of hematoma	2 (3.2%)	6 (3.5%)	0.912
NIHSS scores	14 (10–15)	8 (6–12)	< 0.001
Hematoma volume (ml)	23 (18–28)	14 (10–15)	< 0.001
Blood leucocyte count (× 10^9^/l)	7.9 (4.9–10.5)	6.2 (4.7–8.6)	0.044
Blood glucose levels (mmol/l)	10.4 (8.3–14.3)	9.2 (7.5–11.0)	0.024
Serum ATF3 levels (pg/ml)	184.8 (127.1–229.1)	120.9 (98.8–151.3)	< 0.001

*Notes*: Data were presented as count (percentage), mean ± standard deviation or median (upper‐lower quartiles) as considered suitable. The Chi‐square test, Fisher exact test, Student's 𝑡‐test or Mann‐Whitney test was in use for intergroup comparison.

Abbreviations: NIHSS, National Institutes of Health Stroke Scale; COPD, chronic obstructive pulmonary disease; ATF3, activating transcription factor 3.

**FIGURE 10:**
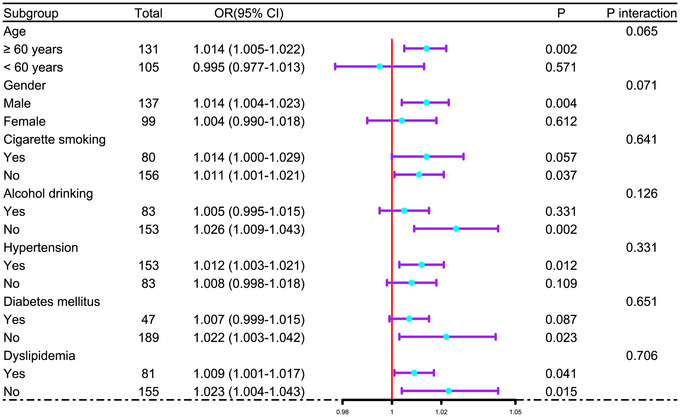
Assessment of interactional effects between the conventional factors and admission serum activating transcription factor three levels in terms of associating with SAP after acute intracerebral hemorrhage. As depicted in the forest graph, age, sex, tobacco smoking, and more had non‐interactional effects with admission serum activating transcription factor three levels in terms of association with SAP according to subgroup analysis and likelihood ratio test (all *p* interaction > 0.05). OR means odds ratio; 95% CI, 95% confidence interval.

**FIGURE 11:**
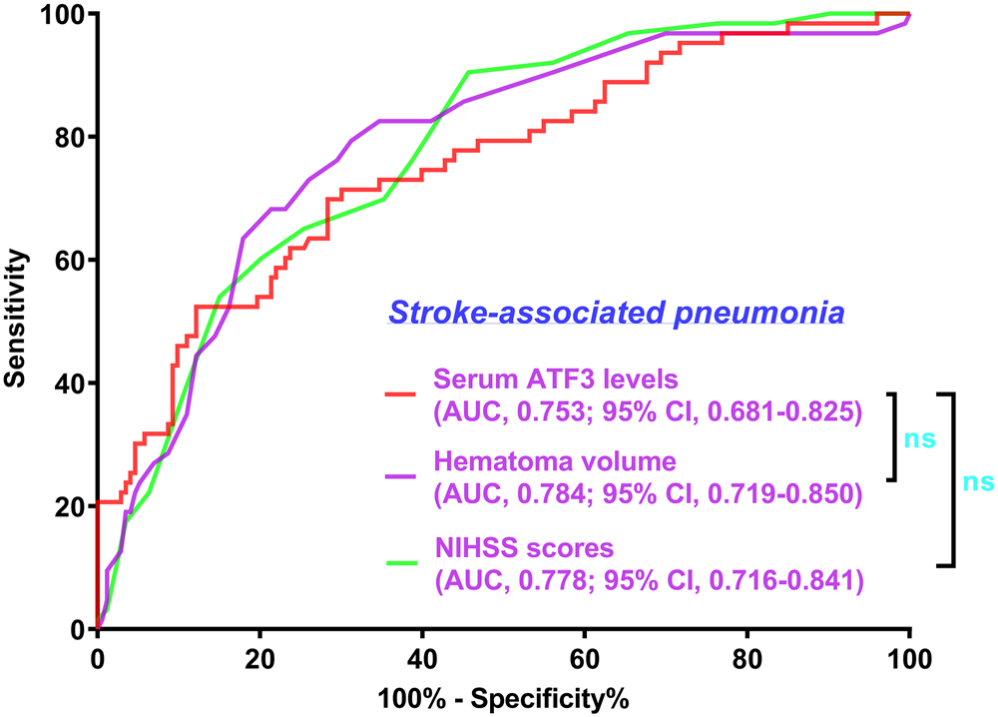
Ability of admission serum activating transcription factor three levels and other variables in anticipation of SAP after acute intracerebral hemorrhage. Under the ROC curve, serum activating transcription factor three levels at admission were strongly predictive of SAP, and its ability resembled those of the NIHSS scores and hematoma volume in patients with acute intracerebral hemorrhage (both *p* > 0.05). ATF3 stands for activating transcription factor 3; NIHSS, National Institutes of Health Stroke Scale; AUC, area under the curve; 95% CI, 95% confidence interval; ns, non‐significant.

Supplementary Figure 13 displays that the E‐value, a component of sensitivity assessment, was 1.111 (95% CI, 1.047–1.149). In Figure 10, the relationship between admission serum ATF3 levels and SAP was not statistically significantly moderated by age, sex, and so on in the setting of subgroup analysis (all *p* > 0.05). Furthermore, for the sake of differentiating individuals with SAP from all patients, the AUC of admission serum ATF3 levels was not substantially different from those of NIHSS scores and hematoma volume (both *p* > 0.05; Figure 11).

## 4 Discussion

As far as we are concerned, this is the first series to proffer some insights into the relevance of serum ATF3 levels to poor clinical outcomes of ICH, covering END, SAP, and poor six‐month prognosis following ICH. Our chief findings are that: (1) serum ATF3 levels were shortly enhanced after ICH, peaked at day 3, and until day 14, were still significantly higher than those of controls; (2) serum ATF3 levels were independently correlated with both NIHSS score and hematoma; (3) as affirmed by a plethora of statistical modalities, serum ATF3 levels robustly anticipated SAP, END, and poor neurological function; and (4) by aid of ROC curve analysis, serum ATF3 levels effectively distinguished risk of SAP, END, or poor prognosis, and its predictive abilities were analogous to those of NIHSS scores and hematoma volume. In summary, serum ATF3 levels may be accepted as an appealing prognostic biomarker of ICH.

ATF3 is fully convinced as a stress‐associated transcription factor and is ubiquitously expressed in neurons and glial cells; and its expression in brain tissues were obviously up‐regulated in reaction to various stressors, including apoptosis, oxidative stress, inflammatory response, and ischemia/reperfusion at the state of spinal cord injury, ischemic stroke, or traumatic brain injury (Holland and Ramer 2023; Katz et al. 2022). Although double‐sided effects have been recently recognized for ATF3 in inflammatory responses; accumulating evidence is highly supportive of the conception that ATF3 may be a protective factor, considering it could potently defend neurons against inflammatory and oxidative injuries in ischemic and traumatized brain tissues (Förstner et al. 2018; Kao et al. 2023; Ma et al. 2022; Wang et al. 2012; Song et al. 2011). Taken together, ATF3 has firmly emerged as a potential therapeutic target in acute brain injury.

Brain injuries are accompanied by various intricate molecular interactions, which involve regional and systemic inflammatory reactions; and systemic damage typified by systemic inflammatory response syndrome is ubiquitous in acute brain injury diseases, including ICH (Skidmore and Andrefsky 2002; Tschoe et al. 2020; Tapia‐Pérez et al. 2016; Liu et al. 2023). Of Note, elevated circulating ATF3 levels have been reported in a previous clinical study of two groups of patients respectively diagnosed with ischemic stroke and spinal cord injury (Pan et al. 2024). In this cohort of individuals with ICH, the median sampling time at admission was roughly nine hours post‐ICH. At this time point, serum ATF3 levels of patients with ICH were substantially higher than those of controls; moreover, they reached a maximum value at day 3 and remained markedly higher up to 14 days post‐ICH. Compelling data have alluded to the point that ATF3 may confer a compensatory or adaptive homeostatic role in alleviating inflammatory responses under inflammation‐related pathological circumstances (Lin and Cheng 2018; Hai et al. 2010). As a consequence, upregulation of ATF3 expression in the central nervous system may be a manifestation of organic self‐repair or healing that is prone to restore cellular homeostasis and even recover neurological functions (Li et al. 2023). Perhaps enhanced circulating ATF3 levels following ICH should be a compensatory action to central and systemic injuries.

Admittedly, NIHSS is fully believed to be a scaling system for reflecting neurological deficits, and hematoma volume is sufficiently accepted as a radiological index for mirroring hemorrhage amount in this ICH setting (Du et al. 2014; Ma et al. 2025). A clinical epidemiological study showed that enhanced blood levels of ATF3 were positively proportional to neurological deficits indicated by admission NIHSS scores on univariate analysis of thirty patients with ischemic stroke (Pan et al. 2024). In our study, the sample number of patients with ICH was enlarged to 236, and 114 of them still allowed for blood drawings at many time points. Not only were admission NIHSS scores and initial hematoma size, via univariate analysis, were correlated with serum ATF3 levels at all blood‐collection time points in patients consenting to serial sampling, but also admission serum ATF3 levels, by multifactorial analysis, were independently related to initial NIHSS scores and hematoma amount in all patients. Overall, our data have supplied evidence to state that serum ATF3 levels may be linked to ICH intensity.

Anticipation of outcomes is an indispensable procedure in clinical practice. The three noteworthy clinical outcomes in ICH‐relevant epidemiological investigations are END, SAP, and poor prognosis, which were currently chosen as the dependent variables in our study. In the first step, patients allowing for serial sampling were regarded as study subjects. AUC was calculated from serum ATF3 levels at admission and subsequently was set as a reference. AUC of serum ATF3 levels at other time points were compared with it, and demonstrably, AUC of admission serum ATF3 levels was not inferior to those at other time points; leading to the inference that serum ATF3 levels at admission may have predictive ability of good clinical value for END, SAP, and poor prognosis subsequent to acute ICH. Consequently, serum‐based ATF3 levels at admission were chosen as an independent variable for further multivariate analysis in all patients. As expected, admission serum‐based ATF3 levels were actually independently associated with END, SAP, and poor prognosis. Moreover, by way of auxiliary statistical approaches, including sensitivity analysis, linearity appraisal, interactional effect investigation, and collinearity analysis, our results turned robust and reliable. In the context of ROC analysis, ATF3 levels at admission were in possession of an AUC, which was comparable to those of baseline NIHSS scores and hematoma volume. Especially, as for sensitivity analysis, besides E value's calculation, both END and SAP, as the two outcome variables, became supplements to poor prognosis, altogether proving that serum ATF3 may be firmly related to poor clinical outcomes. In a word, these data adequately support the viewpoint that serum ATF3 may be a useful adjunct for predicting poor clinical outcomes in ICH.

The strengths and limitations of this study warrant consideration. The strengths are that: (1) as far as we are aware, this study was designed to do the first measurement to ATF3 levels in biofluid samples of patients with acute ICH, and the subsequent finding was that serum ATF3 may reliably become a potential prognostic biochemical metric of human ICH; and (2) so as to fully connect serum ATF3 with several clinical outcomes of ICH, including SAP, END, and poor prognosis, not only several multivariate models but also various auxiliary statistical avenues were adopted, thereby forming more scientific and rational conclusions. The limitations are that: (1) only cases diagnosed with supratentorial intraparenchymal bleeding were inclusive in the present study, and thus, whether serum ATF3 embodies similar prognostic value in primary intraventricular hemorrhage or subtentorial bleeding needs to be further verified; and (2) according to statistics, a sufficient sample size was calculated here for carrying out this clinical cohort study; nonetheless, it is paramount of repetitive validation of the results in larger cohort studies ahead of generalization.

## 5 Conclusions

Serum ATF3 levels escalate rapidly following acute ICH and remain higher than in normal individuals up to at least 14 days post‐ICH. NIHSS scores in conjunction with hematoma volume are firmly correlated with serum ATF3 levels. Serum ATF3 levels are independently associated with SAP, END, and six‐month poor neurological status post‐ICH, along with satisfactory predictive efficiency. Suggestively, serum ATF3 level could be used as an alternative biomarker of prognostic prediction in ICH.

## Author Contributions

**Yingrui Gu**: conceptualization, investigation, methodology, validation, formal analysis, software, project administration, resources, supervision, writing–original draft, writing–review and editing. **Xin Wang**: conceptualization, investigation, writing–original draft, writing–review and editing, project administration, resources, supervision, data curation, software, methodology, validation. **Peng Xu**: investigation, conceptualization, methodology, validation, writing–review and editing, writing–original draft. **Zhiyong Li**: conceptualization, investigation, writing–original draft, writing–review and editing, project administration, formal analysis. **Xiaodong Deng**: conceptualization, investigation, funding acquisition, writing–original draft, writing–review and editing, visualization, validation, project administration, formal analysis, supervision. **Yong Cui**: resources, supervision, data curation, software, formal analysis, project administration, writing–review and editing, writing–original draft, investigation, conceptualization, methodology, validation.

Yingrui Gu, Xin Wang and Peng Xu contributed equally to this work.

## Funding

This work is supported by Pudong New Area Demonstration Project for Inheritance and Innovation of Traditional Chinese Medicine (No. YC‐2023‐0201)

## Conflicts of Interest

The authors declare no conflicts of interest.

## Supporting information




**Supporting Table: 1** Bivariate correlation analysis in patients willing for serial blood drawings subsequent to acute ICH.
**Supporting Table 2**: Serum ATF3 levels among subgroups defined by mRS in patients permitting for blood drawings at multiple time points following acute ICH.
**Supporting Table 3**: Serum ATF3 levels and its area under ROC curve for poor prognosis in patients volunteering for multiple‐time sampling following acute ICH.
**Supporting Table 4**: Serum ATF3 levels and its area under ROC curve for neurological deterioration in patients consenting for multiple‐time sampling following acute ICH.
**Supporting Table 5**: Serum ATF3 levels and its area under ROC curve for SAP in patients accepting for multiple‐time sampling following acute ICH.**Supplementary Figure 1** Serum ATF3 levels at admission and NIHSS of patients diseased of acute ICH.
**Supporting Fig.2**: Serum ATF3 levels at admission and hematoma volume of patients following acute ICH.
**Supporting Fig.3**: Serum ATF3 levels at admission and six‐month mRS scores post‐acute ICH.
**Supporting Fig.4**: Serum ATF3 levels at admission among patients with disparate six‐month mRS scores following acute ICH.
**Supporting Fig.5**: Serum ATF3 levels at admission between patients with poor prognosis and those without the same event six months following acute ICH.
**Supporting Fig.6**: Efficiency with respect to admission serum ATF3 levels in discriminating risk of poor prognosis six months after acute ICH.
**Supporting Fig.7**: Sensitivity appraisal as regards robustness of prognosis association following acute ICH.
**Supporting Fig.8**: Serum ATF3 levels at admission between patients presenting with END and those without the complication after acute ICH.
**Supporting Fig.9**: Anticipation efficiency of admission serum ATF3 levels for early neurological deterioration after acute ICH.
**Supporting Fig.10**: Sensitivity analysis as for robustness of END association after acute ICH.
**Supporting Fig.11**: Serum ATF3 levels at admission between patients with development of SAP and those without the event after acute ICH.
**Supporting Fig.12**: Predictive effectiveness of admission serum ATF3 levels for SAP following acute ICH.
**Supporting Fig.13**: Sensitivity analysis showing robustness of associating with SAP after acute ICH.

## Data Availability

The datasets generated and/or analyzed during the current study are not publicly available because of the inclusion of personal data but can be obtained from the corresponding author upon reasonable request.
