# Supplementary material for: Practicability of Activating Transcription Factor 3 as a Serological Marker for Severity Appraisal and Outcome Anticipation in Acute Supratentorial Intracerebral Hemorrhage: A Two‐center Observational Analytical Study
Source: Brain Behav. 2025 Nov 17;15(11):e71070. doi: 10.1002/brb3.71070 (PMC12623450; doi:10.1002/brb3.71070)
Supplement: Supplementary file 1 — Supporting Table: 1 Bivariate correlation analysis in patients willing for serial blood drawings subsequent to acute ICH. Supporting Table 2: Serum ATF3 levels among subgroups defined by mRS in patients permitting for blood drawings at multiple time points following acute ICH. Supporting Table 3: Serum ATF3 levels and its area under ROC curve for poor prognosis in patients volunteering for multiple‐time sampling following acute ICH. Supporting Table 4: Serum ATF3 levels and its area under ROC curve for neurological deterioration in patients consenting for multiple‐time sampling following acute ICH. Supporting Table 5: Serum ATF3 levels and its area under ROC curve for SAP in patients accepting for multiple‐time sampling following acute ICH.Supplementary Figure 1 Serum ATF3 levels at admission and NIHSS of patients diseased of acute ICH. Supporting Fig.2: Serum ATF3 levels at admission and hematoma volume of patients following acute ICH. Supporting Fig.3: Serum ATF3 levels at admission and six‐month mRS scores post‐acute ICH. Supporting Fig.4: Serum ATF3 levels at admission among patients with disparate six‐month mRS scores following acute ICH. Supporting Fig.5: Serum ATF3 levels at admission between patients with poor prognosis and those without the same event six months following acute ICH. Supporting Fig.6: Efficiency with respect to admission serum ATF3 levels in discriminating risk of poor prognosis six months after acute ICH. Supporting Fig.7: Sensitivity appraisal as regards robustness of prognosis association following acute ICH. Supporting Fig.8: Serum ATF3 levels at admission between patients presenting with END and those without the complication after acute ICH. Supporting Fig.9: Anticipation efficiency of admission serum ATF3 levels for early neurological deterioration after acute ICH. Supporting Fig.10: Sensitivity analysis as for robustness of END association after acute ICH. Supporting Fig.11: Serum ATF3 levels at admission between patients with develo [file BRB3-15-e71070-s001.docx]

Supplementary Table 1 Bivariate correlation analysis in patients willing for serial blood drawings subsequent to acute intracerebral hemorrhage

| Sampling time | NIHSS scores | | Hematoma volume | | mRS scores | |
| --- | --- | --- | --- | --- | --- | --- |
|  | ρ | P values | ρ | P values | ρ | P values |
| Admission | 0.586 | <0.001 | 0.583 | <0.001 | 0.632 | <0.001 |
| Day 1 | 0.632 | <0.001 | 0.601 | <0.001 | 0.616 | <0.001 |
| Day 3 | 0.619 | <0.001 | 0.613 | <0.001 | 0.655 | <0.001 |
| Day 5 | 0.536 | <0.001 | 0.516 | <0.001 | 0.599 | <0.001 |
| Day 7 | 0.526 | <0.001 | 0.540 | <0.001 | 0.586 | <0.001 |
| Day 10 | 0.496 | <0.001 | 0.494 | <0.001 | 0.587 | <0.001 |
| Day 14 | 0.517 | <0.001 | 0.509 | <0.001 | 0.594 | <0.001 |

Correlation was assessed in use of the Spearman test. NIHSS stands for National Institutes of Health Stroke Scale; ATF3, activating transcription factor 3; mRS, modified Rankin Scale; ICH, intracerebral hemorrhage.

Supplementary Table 2 Serum activating transcription factor 3 levels among subgroups defined by modified Rankin Scale in patients permitting for blood drawings at multiple time points following acute intracerebral hemorrhage

| Sampling time | Modified Rankin Scale scores | | | | | | | P values |
| --- | --- | --- | --- | --- | --- | --- | --- | --- |
|  | 0 | 1 | 2 | 3 | 4 | 5 | 6 |  |
| Admission | 71.0 (67.7-72.6) | 115.0 (92.2-128.1) | 121.0 (101.6-153.6) | 104.6 (88.8-179.5) | 164.0 (126.2-185.0) | 201.7 (170.4-239.3) | 204.0 (175.8-216.2) | <0.001 |
| Day 1 | 47.7 (36.2-57.7) | 151.5 (84.7-290.3) | 249.6 (114.9-348.6) | 149.3 (111.6-402.9) | 350.7 (240.9-360.6) | 371.4 (354.6-503.6) | 360.8 (351.6-365.4) | <0.001 |
| Day 3 | 83.3 (68.4-121.3) | 283.7 (135.4-626.6) | 654.9 (211.9-928.2) | 295.7 (188.7-1030.6) | 996.1 (660.5-1050.6) | 1158.2 (992.5-1282.8) | 1138.6 (1022.3-1203.1) | <0.001 |
| Day 5 | 98.0 (70.1-121.6) | 313.9 (179.3-399.1) | 330.5 (197.2-478.9) | 219.4 (180.4-487.9) | 485.9 (387.8-492.1) | 578.3 (468.7-607.3) | 559.7 (498.3-588.3) | <0.001 |
| Day 7 | 57.7 (40.2-76.9) | 162.0 (108.7-168.7) | 163.2 (141.2-205.2) | 139.2 (100.2-238.3) | 221.5 (162.8-244.8) | 256.7 (217.3-298.5) | 284.1 (229.0-289.0) | <0.001 |
| Day 10 | 33.1 (17.9-40.3) | 101.3 (52.2-109.1) | 105.7 (70.9-131.3) | 85.4 (44.6-100.3) | 133.3 (111.5-139.1) | 140.5 (116.9-150.9) | 144.3 (133.9-156.0) | <0.001 |
| Day 14 | 21.8 (18.0-24.9) | 44.5 (27.8-64.3) | 50.7 (30.6-94.9) | 31.5 (26.0-35.1) | 100.8 (60.5-104.2) | 106.1 (95.2-113.9) | 106.8 (103.2-107.1) | <0.001 |

Variables were presented as median (upper-lower quartiles), with the Kruskal-Wallis H test for statistical analysis.

Supplementary Table 3 Serum activating transcription factor 3 levels and its area under receiver operating characteristic curve for poor prognosis in patients volunteering for multiple-time sampling following acute intracerebral hemorrhage

| Sampling time | Six-month neurological outcome | | | ROC curve analysis | |
| --- | --- | --- | --- | --- | --- |
|  | Poor prognosis | Good prognosis | P values | AUC (95% CI) | P values |
| Admission | 166.1 (113.6-207.9) | 111.6 (89.1-134.3) | <0.001 | 0.767 (0.679-0.856) | Reference |
| Day 1 | 353.1 (229.6-386.4) | 130.4 (79.3-323.2) | <0.001 | 0.773 (0.687-0.860) | 0.793 |
| Day 3 | 1013.2 (455.0-1194.9) | 299.1 (148.4-777.4) | <0.001 | 0.790 (0.707-0.874) | 0.086 |
| Day 5 | 485.9 (299.9-575.2) | 268.3 (180.0-441.2) | <0.001 | 0.752 (0.662-0.841) | 0.291 |
| Day 7 | 229.0 (157.3-281.5) | 152.7 (105.9-174.2) | <0.001 | 0.744 (0.651-0.837) | 0.255 |
| Day 10 | 133.4 (92.3-145.4) | 99.5 (44.4-117.0) | <0.001 | 0.740 (0.648-0.831) | 0.209 |
| Day 14 | 98.5 (37.2-106.4) | 37.5 (25.9-73.8) | <0.001 | 0.729 (0.636-0.822) | 0.037 |

Serum activating transcription factor 3 levels were presented as median (upper-lower quartiles), with the Mann-Whitney test as statistical method. The Z test was done to achieve comparison in area under curve. AUC indicates area under curve; 95% CI, 95% confidence interval; ROC, receiver operating characteristic.

Supplementary Table 4 Serum activating transcription factor 3 levels and its area under receiver operating characteristic curve for neurological deterioration in patients consenting for multiple-time sampling following acute intracerebral hemorrhage

| Sampling time | Neurological deterioration | | | ROC curve analysis | |
| --- | --- | --- | --- | --- | --- |
|  | Existence | Non-existence | P values | AUC (95% CI) | P values |
| Admission | 170.6 (123.1-225.8) | 114.8 (89.1-159.1) | <0.001 | 0.744 (0.644-0.845) | Reference |
| Day 1 | 363.3 (290.7-415.6) | 220.9 (81.3-349.3) | <0.001 | 0.751 (0.655-0.847) | 0.813 |
| Day 3 | 959.3 (733.7-1238.5) | 412.5 (171.3-974.5) | <0.001 | 0.756 (0.659-0.854) | 0.515 |
| Day 5 | 483.5 (304.8-585.5) | 301.7 (188.0-478.6) | <0.001 | 0.730 (0.628-0.833) | 0.461 |
| Day 7 | 210.8 (156.4-295.7) | 158.2 (106.5-214.0) | <0.001 | 0.707 (0.601-0.814) | 0.195 |
| Day 10 | 124.6 (87.8-150.4) | 101.3 (44.6-132.1) | 0.004 | 0.670 (0.563-0.777) | 0.023 |
| Day 14 | 85.8 (36.2-108.3) | 45.4 (26.4-94.9) | 0.001 | 0.687 (0.582-0.792) | 0.034 |

Serum activating transcription factor 3 levels were presented as median (upper-lower quartiles), with the Mann-Whitney test as statistical method. The Z test was done to finish comparison in area under curve. AUC indicates area under curve; 95% CI, 95% confidence interval; ROC, receiver operating characteristic.

Supplementary Table 5 Serum activating transcription factor 3 levels and its area under receiver operating characteristic curve for stroke-associated pneumonia in patients accepting for multiple-time sampling following acute intracerebral hemorrhage

| Sampling time | Stroke-associated pneumonia | | | ROC curve analysis | |
| --- | --- | --- | --- | --- | --- |
|  | Presence | Absence | P values | AUC (95% CI) | P values |
| Admission | 161.7 (128.9-208.7) | 114.8 (90.8-158.4) | <0.001 | 0.728 (0.624-0.832) | Reference |
| Day 1 | 354.4 (328.4-422.1) | 220.9 (85.7-351.6) | <0.001 | 0.742 (0.641-0.843) | 0.594 |
| Day 3 | 985.3 (794.7-1199.3) | 412.5 (171.3-957.1) | <0.001 | 0.753 (0.658-0.848) | 0.211 |
| Day 5 | 480.2 (359.4-553.6) | 301.7 (190.0-478.7) | 0.001 | 0.704 (0.597-0.812) | 0.218 |
| Day 7 | 217.3 (164.0-274.8) | 157.6 (106.8-207.8) | 0.001 | 0.698 (0.588-0.807) | 0.349 |
| Day 10 | 132.3 (99.9-147.1) | 101.3 (49.3-126.4) | 0.002 | 0.690 (0.581-0.800) | 0.250 |
| Day 14 | 91.6 (42.1-105.1) | 45.5 (26.4-94.9) | 0.003 | 0.677 (0.571-0.784) | 0.100 |

Serum activating transcription factor 3 levels were presented as median (upper-lower quartiles), with the Mann-Whitney test as statistical method. The Z test was done to complete comparison in area under curve. AUC indicates area under curve; 95% CI, 95% confidence interval; ROC, receiver operating characteristic.

# Figure Legends

**Supplementary Figure 1**

Serum activating transcription factor 3 levels at admission and National Institutes of Health Stroke Scale scores of patients diseased of acute intracerebral hemorrhage.

In this scatter graph, there was a significant correlation of admission serum activating transcription factor 3 levels with baseline National Institutes of Health Stroke Scale scores post-acute intracerebral hemorrhage (P<0.001).

ATF3 means activating transcription factor 3; NIHSS, National Institutes of Health Stroke Scale.

**Supplementary Figure 2**

Serum activating transcription factor 3 levels at admission and hematoma volume of patients following acute intracerebral hemorrhage.

In this scatter graph, a marked correlation was revealed between serum levels of activating transcription factor 3 at admission and initial hematoma volume following acute intracerebral hemorrhage (P<0.001).

ATF3 signifies activating transcription factor 3.

**Supplementary Figure 3**

Serum activating transcription factor 3 levels at admission and six-month modified Rankin Scale scores post-acute intracerebral hemorrhage.

In this scatter plot, serum activating transcription factor 3 levels at admission were strongly correlated with modified Rankin Scale scores six months after acute intracerebral hemorrhage (P<0.001).

ATF3 indicates activating transcription factor 3; mRS, modified Rankin Scale.

**Supplementary Figure 4**

Serum activating transcription factor 3 levels at admission among patients with disparate six-month modified Rankin Scale scores following acute intracerebral hemorrhage.

In this violin graph for multiple-group comparisons, serum activating transcription factor 3 levels at admission were lowest in cases with the modified Rankin Scale score of 0 at six-month mark after acute intracerebral hemorrhage, gradually increased from those with the score from 1 to 5, and were highest in those with the score of 6 (P<0.001).

ATF3 denotes activating transcription factor 3; mRS, modified Rankin Scale.

**Supplementary Figure 5**

Serum activating transcription factor 3 levels at admission between patients with poor prognosis and those without the same event six months following acute intracerebral hemorrhage.

In the violin plot for two-group comparison, patients presenting with poor prognosis, relative to those without, displayed profoundly elevated serum activating transcription factor 3 levels at admission (P<0.001).

ATF3 represents activating transcription factor 3.

**Supplementary Figure 6**

Efficiency with respect to admission serum activating transcription factor 3 levels in discriminating risk of poor prognosis six months after acute intracerebral hemorrhage.

Under the receiver operating characteristic curve analysis, admission serum activating transcription factor 3 levels were effectively predictive of poor prognosis after acute intracerebral hemorrhage, the optimal threshold value being generated using the Youden approach.

AUC denotes area under curve; 95% CI, 95% confidence interval. Red circle means cutoff value.

**Supplementary Figure 7**

Sensitivity appraisal as regards robustness of prognosis association following acute intracerebral hemorrhage.

The E-value was calculated based on the odds ratio value of the association between serum activating transcription factor 3 levels at admission and poor prognosis following acute intracerebral hemorrhage, and this value surpassed the odds ratio value, indicating that the prognostic association was relatively robust.

**Supplementary Figure 8**

Serum activating transcription factor 3 levels at admission between patients presenting with early neurological deterioration and those without the complication after acute intracerebral hemorrhage.

As described in the violin graph, patients with early neurological deterioration, as opposed to the remainders, exhibited markedly enhanced serum levels of activating transcription factor 3 at admission (P<0.001).

ATF3 represents activating transcription factor 3; END, early neurological deterioration.

**Supplementary Figure 9**

Anticipation efficiency of admission serum activating transcription factor 3 levels for early neurological deterioration after acute intracerebral hemorrhage.

In the milieu of receiver operating characteristic curve analysis, early neurological deterioration was effectively predicted by admission serum activating transcription factor 3 levels after acute intracerebral hemorrhage, alongside yielding the appropriate cutoff value by applying the Youden method.

AUC denotes area under curve; 95% CI, 95% confidence interval. Red circle means cutoff value.

**Supplementary Figure 10**

Sensitivity analysis as for robustness of early neurological deterioration association after acute intracerebral hemorrhage.

The E-value, as estimated from the odds ratio value, exceeded the odds ratio value, signifying that the association between admission serum levels of activating transcription factor 3 and early neurological deterioration following acute intracerebral hemorrhage was robust.

**Supplementary Figure 11**

Serum activating transcription factor 3 levels at admission between patients with development of stroke-associated pneumonia and those without the event after acute intracerebral hemorrhage.

As shown in the violin graph, patients with stroke-associated pneumonia had notably higher admission serum levels of activating transcription factor 3 than the other remainders (P<0.001).

ATF3 means activating transcription factor 3; SAP, stroke-associated pneumonia.

**Supplementary Figure 12**

Predictive effectiveness of admission serum activating transcription factor 3 levels for stroke-associated pneumonia following acute intracerebral hemorrhage.

Under the receiver operating characteristic curve, stroke-associated pneumonia was efficaciously predicted via serum activating transcription factor 3 levels at admission of patients with acute intracerebral hemorrhage, and the suitable cut-off value was generated by using the Youden method.

AUC denotes area under curve; 95% CI, 95% confidence interval. Red circle means cutoff value.

**Supplementary Figure 13**

Sensitivity analysis showing robustness of associating with stroke-associated pneumonia after acute intracerebral hemorrhage.

The E-value, which was estimated from the odds ratio value, was above the odds ratio value, indicating that the association between admission serum activating transcription factor 3 levels and stroke-associated pneumonia post-acute intracerebral hemorrhage was comparatively strong.


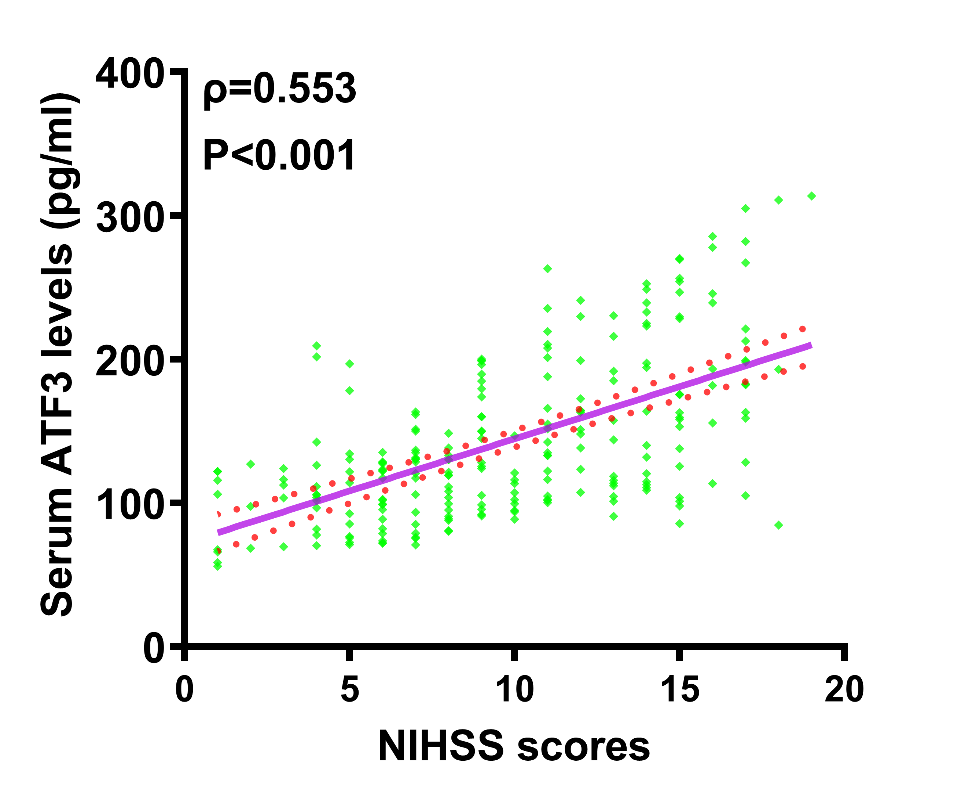


**Supplementary Figure 1**


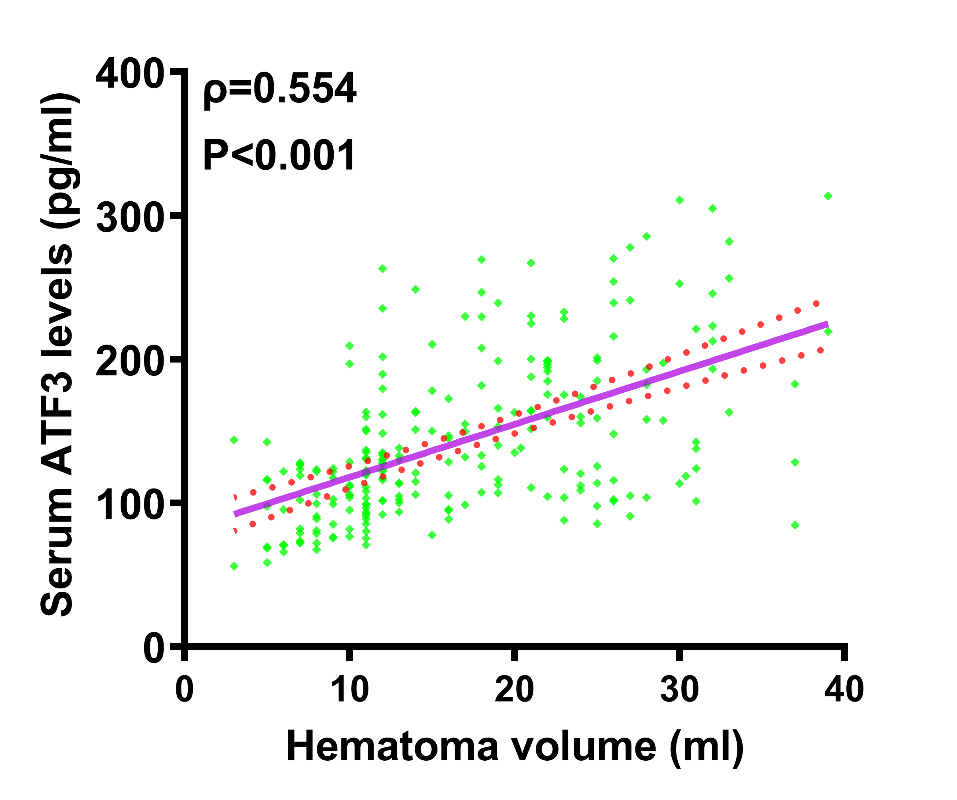


**Supplementary Figure 2**


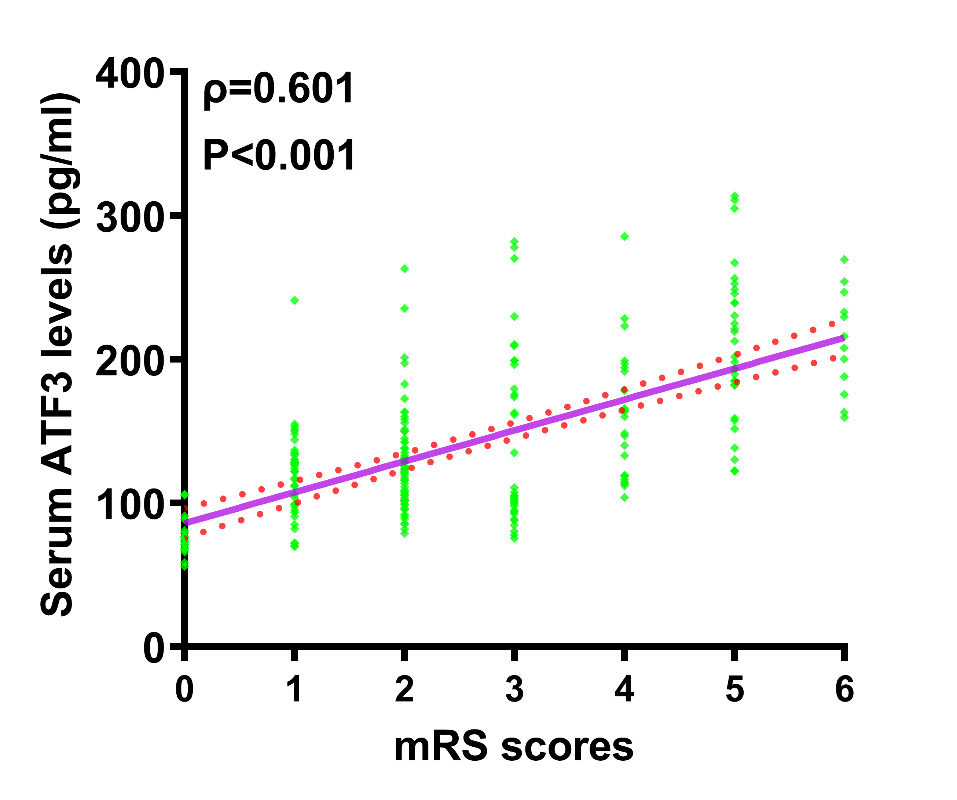


**Supplementary Figure 3**


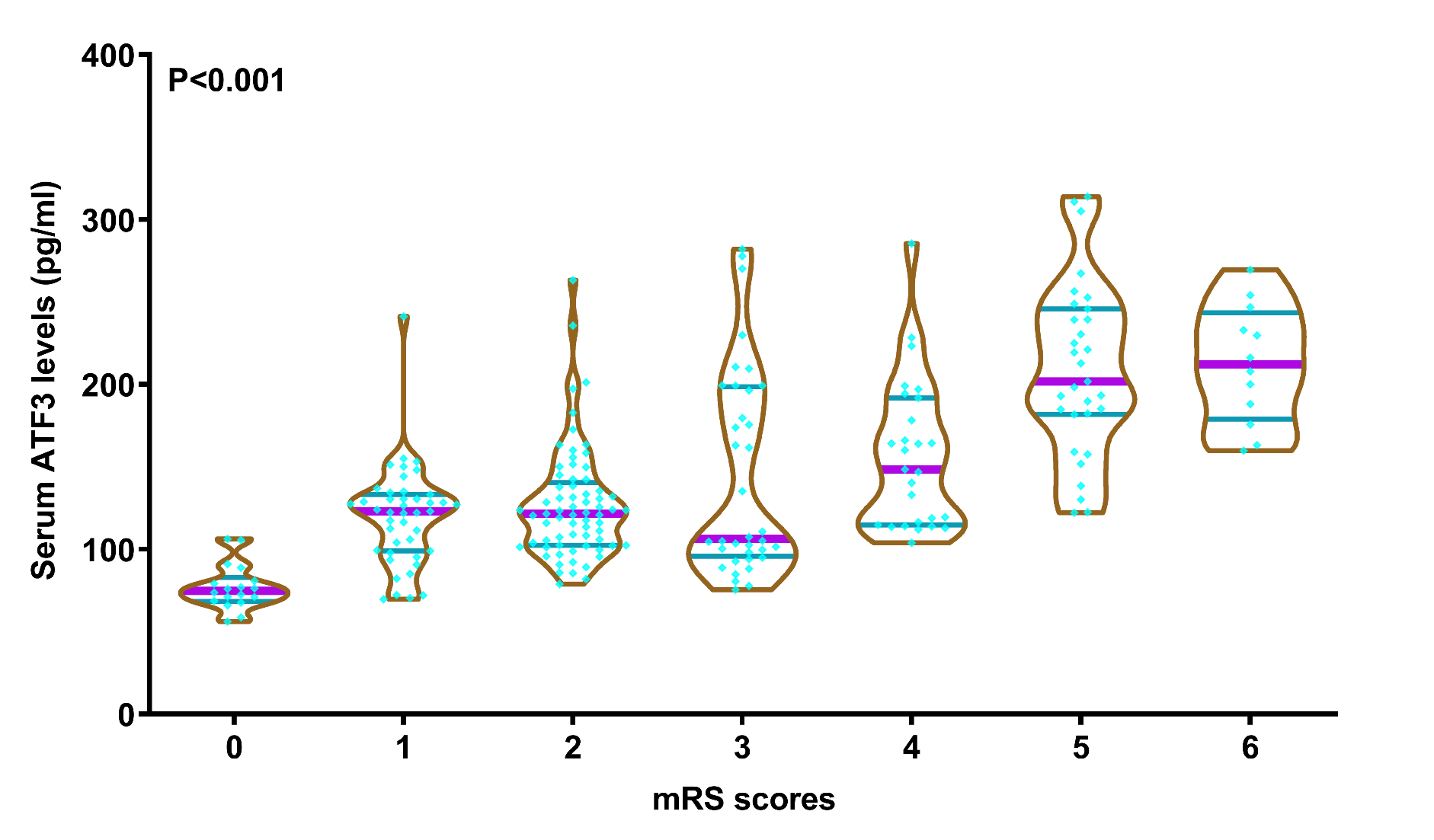


**Supplementary Figure 4**


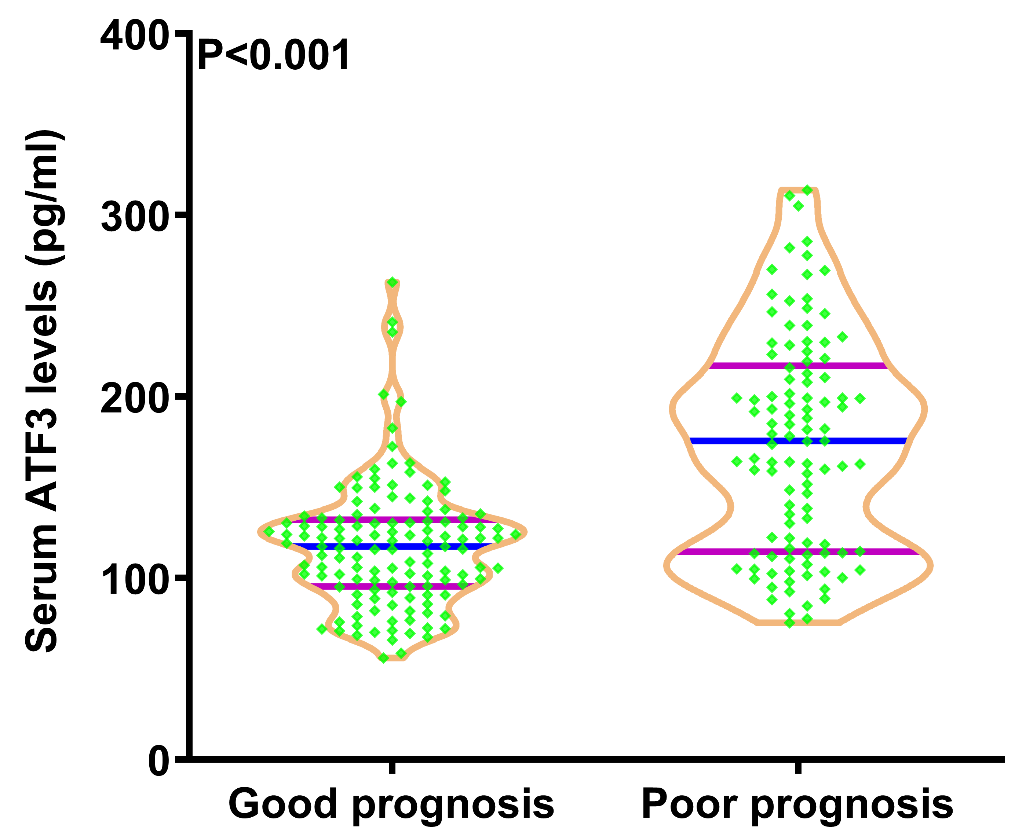


**Supplementary Figure 5**


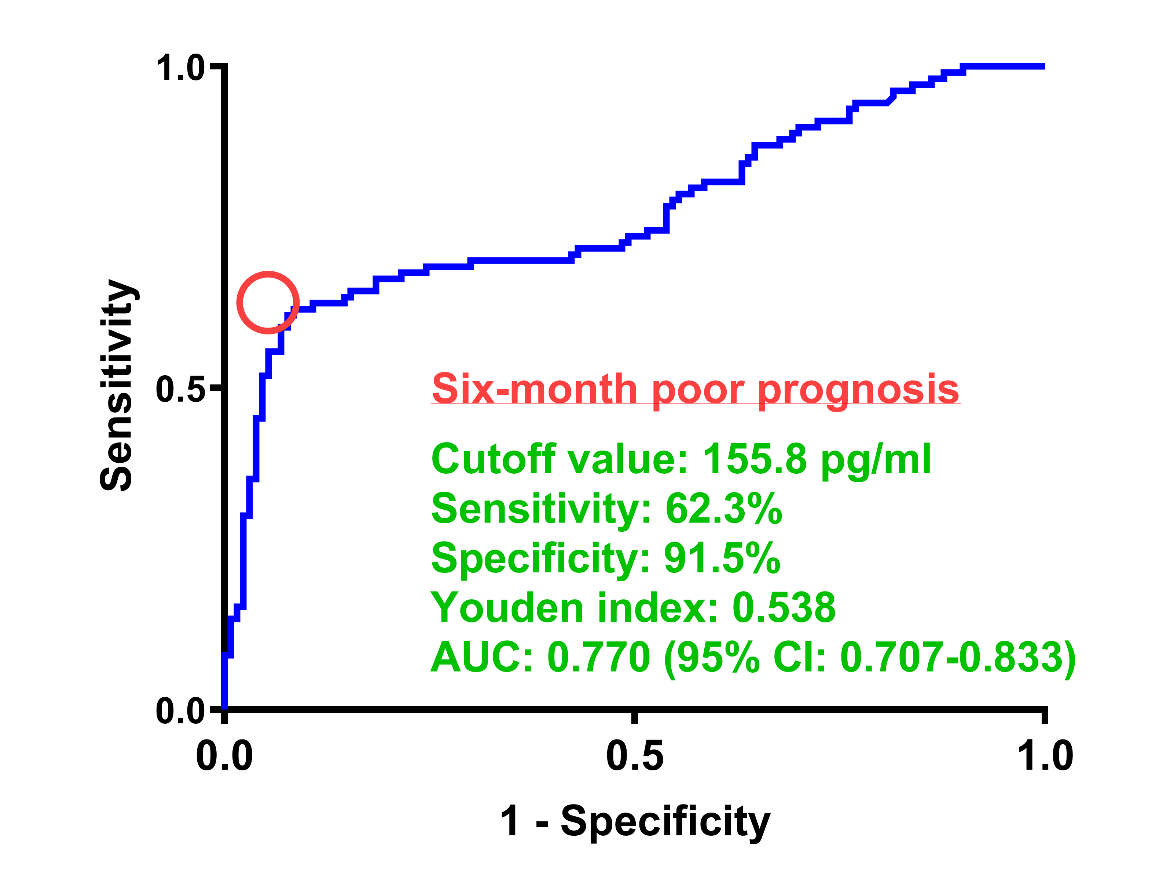


**Supplementary Figure 6**


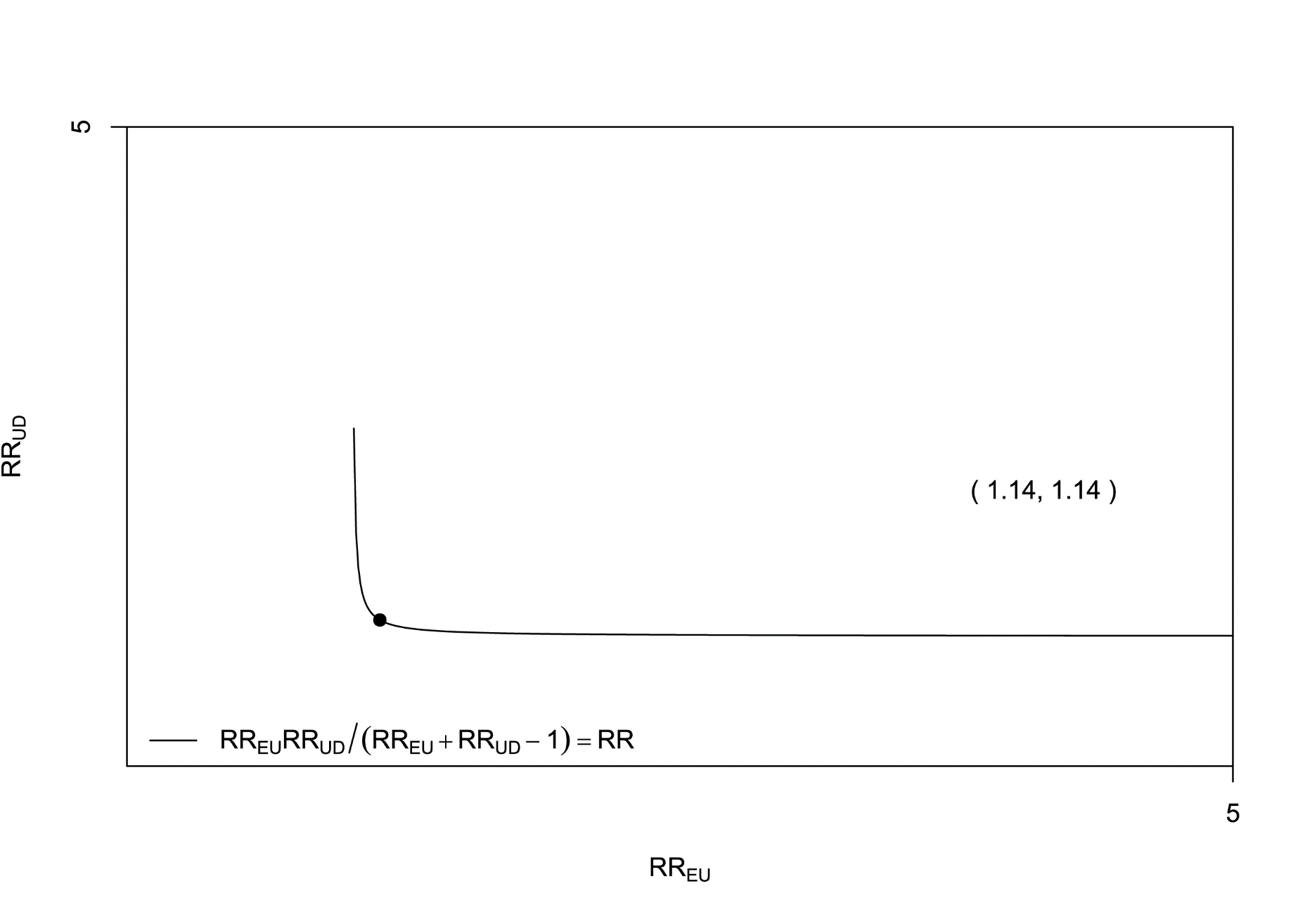


**Supplementary Figure 7**


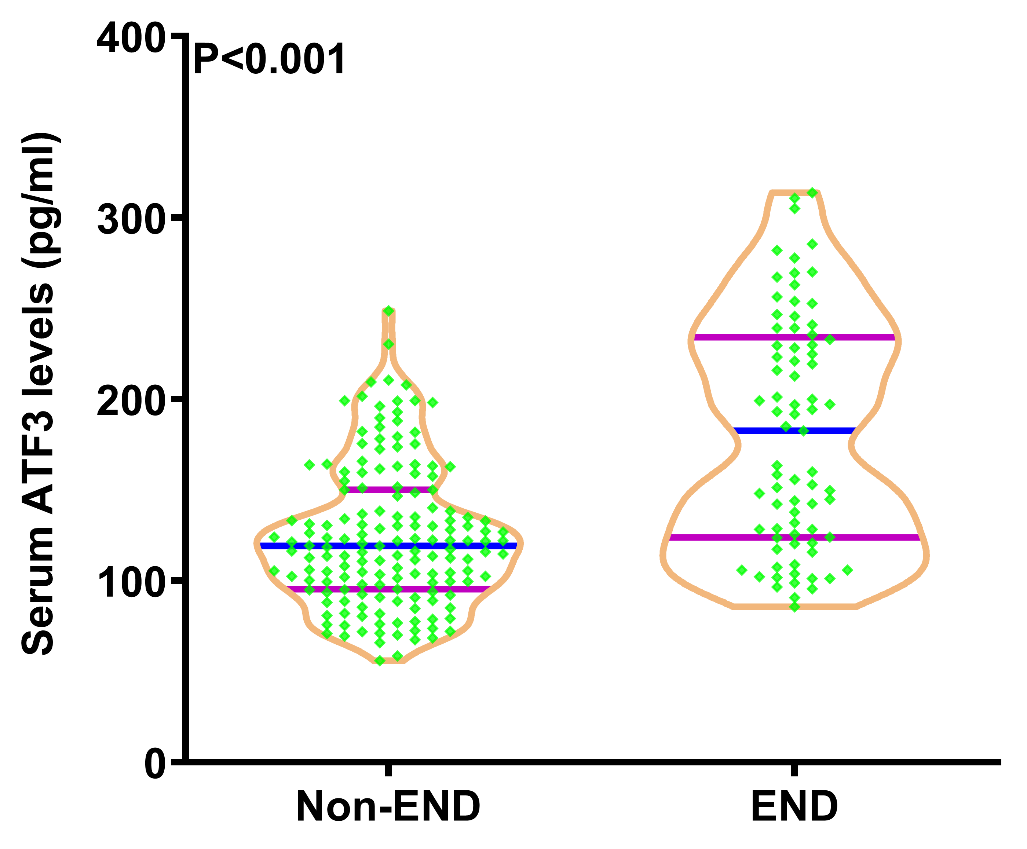


**Supplementary Figure 8**


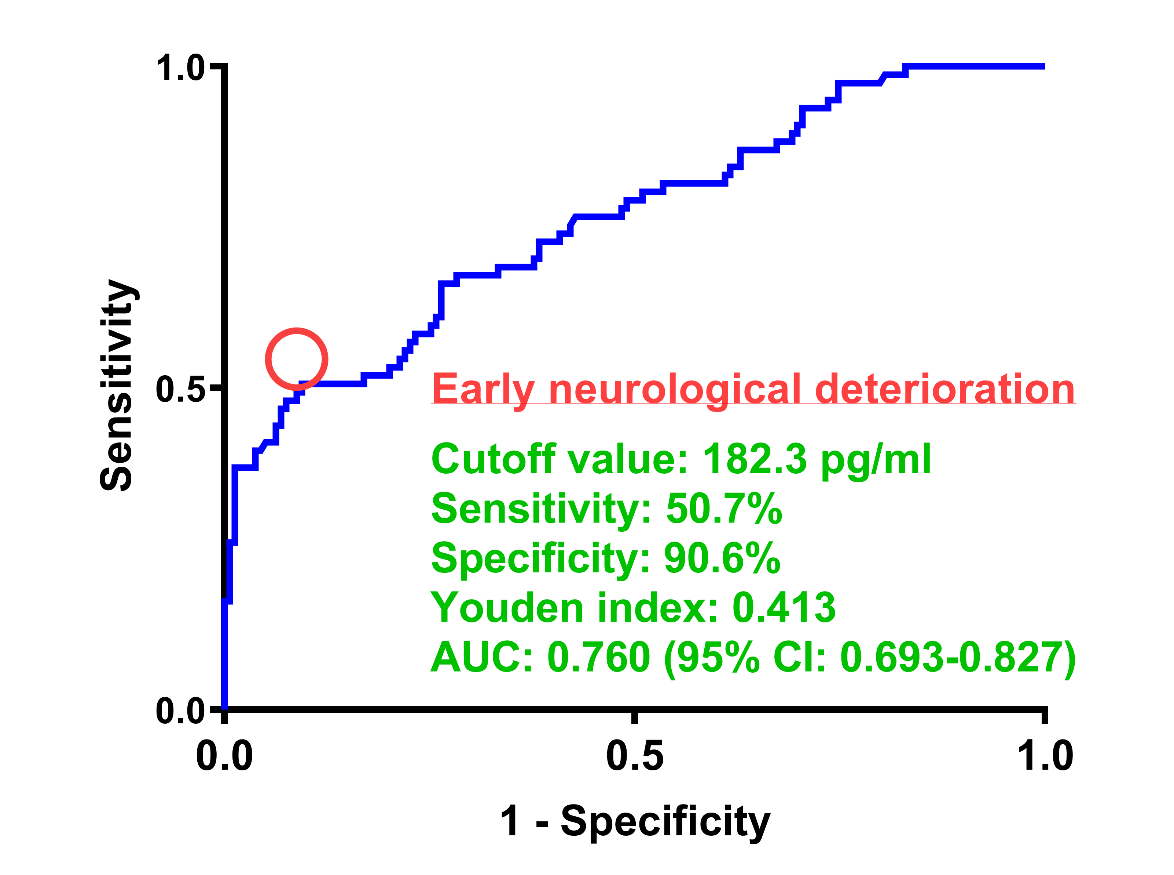


**Supplementary Figure 9**


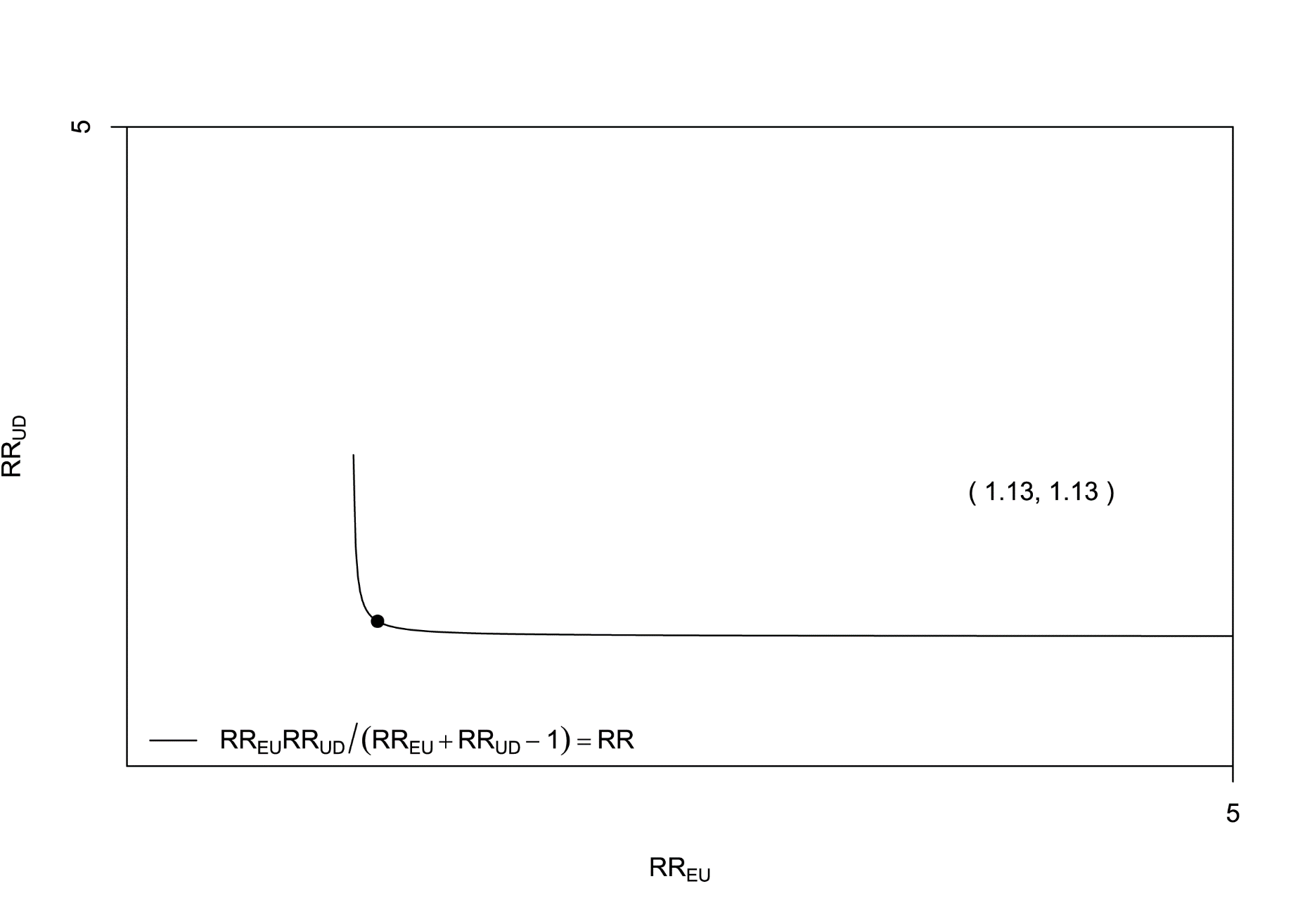


**Supplementary Figure 10**


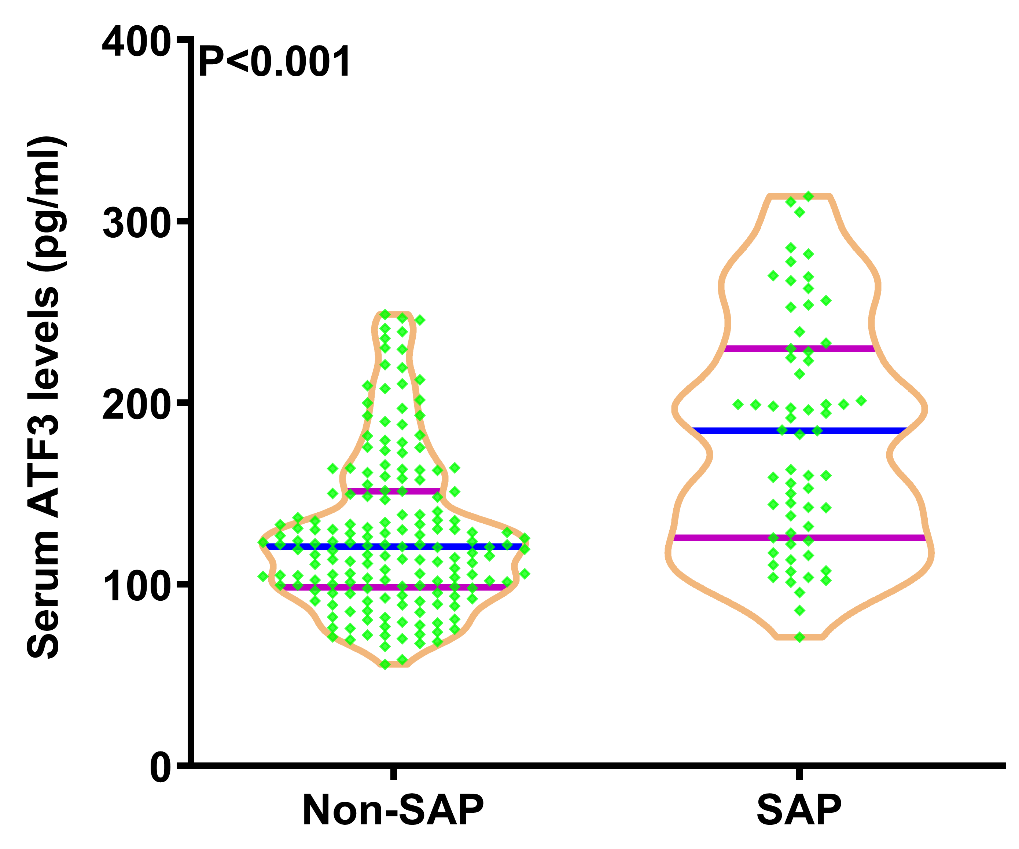


**Supplementary Figure 11**


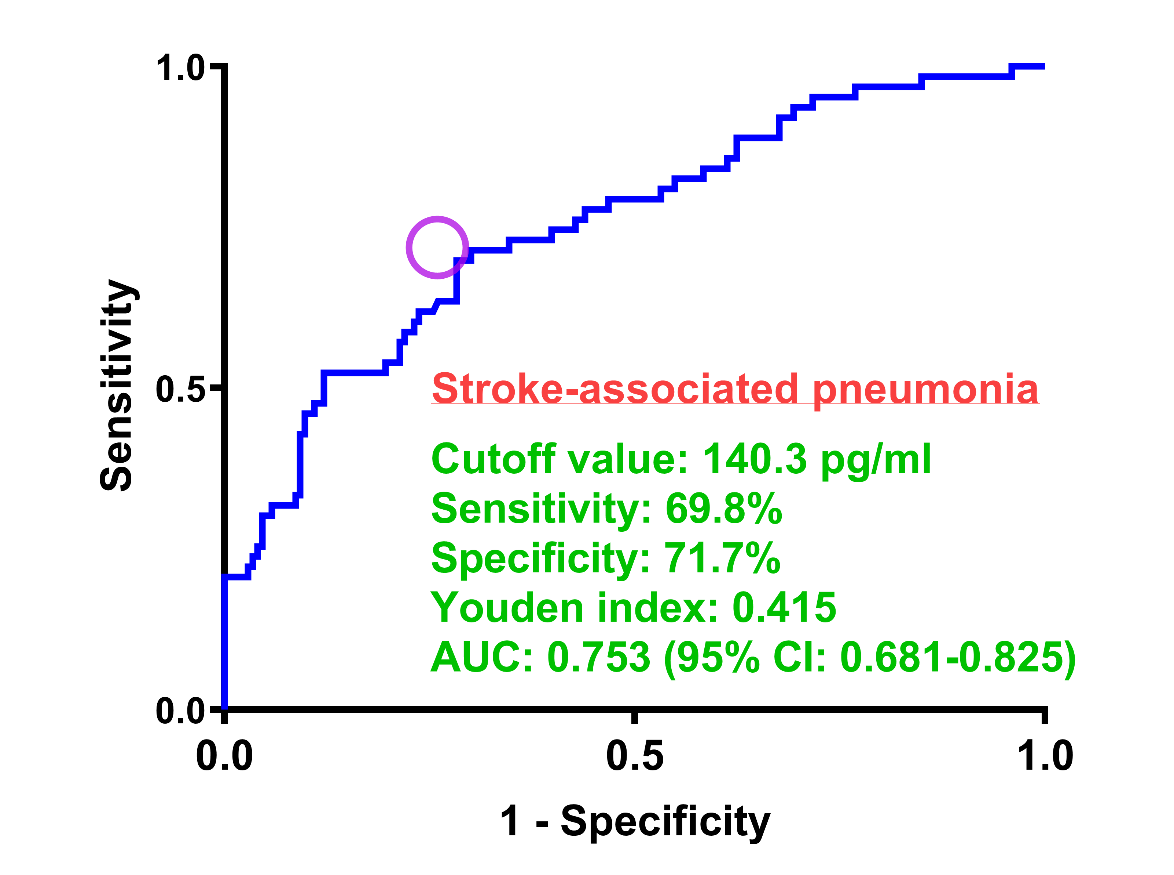


**Supplementary Figure 12**


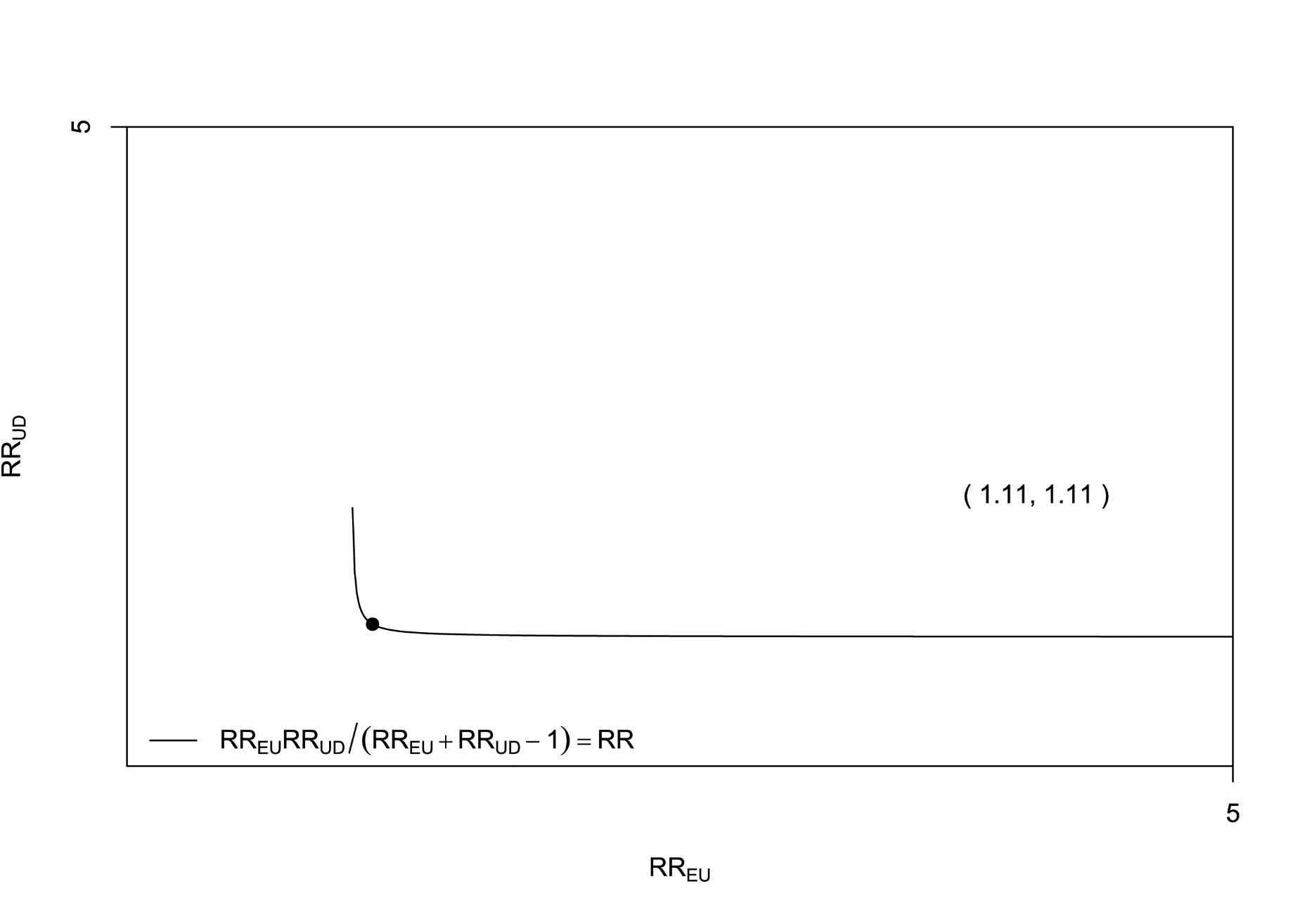


**Supplementary Figure 13**
